# A Co-produced International Qualitative Systematic Review on Lived Experiences of Trauma During Homelessness in Adulthood and Impacts on Mental Health

**DOI:** 10.1177/15248380241286839

**Published:** 2024-11-06

**Authors:** Emma A Adams, Kerry Brennan-Tovey, Joanne McGrath, Steven Thirkle, Neha Jain, Maria Raisa Jessica Aquino, Victoria Bartle, Joanne Kennedy, Margaret Ogden, Jeff Parker, Sophie Koehne, Eileen Kaner, Sheena E Ramsay

**Affiliations:** 1 Newcastle University, UK; 2 Northumbria University, London, UK; 3 Lived Experience, UK; 4 Crisis Skylight Newcastle, UK; 5 Crisis, London, UK; 6 Pathway, London, UK

**Keywords:** alcohol and drugs, mental health, qualitative, trauma

## Abstract

Trauma can be both a cause and a consequence of homelessness and has lasting impacts on mental health and wellbeing. Often research focusses on trauma and adversity in childhood leading to homelessness, but understanding traumatic experiences during adulthood homelessness can be just as important for informing intervention development and policies to mitigate and eradicate homelessness. Working with people with lived experience of homelessness, this review aimed to synthesis the qualitative evidence exploring the impact of trauma during homelessness on mental health (including substance use) from the perspective of adults (18 years of age and older) experiencing homelessness. Alongside gray literature, ASSIA, CINAHL, Cochrane, EMBASE, MEDLINE, Proquest theses and dissertations, PsychInfo, Scopus and Web of Science were searched from inception until February 2024. No language, date, or geographical limits were applied. A ‘best-fit’ framework synthesis of 26 papers, covering the experience of over 900 people, identified three overarching themes linked with the SAMHSA three E’s of trauma: 1) making sense of homelessness as a trauma, 2) dealing with the impacts of trauma and 3) responses to repeated exposure to trauma. Trauma rarely takes place in isolation and often prior experiences shape how people experiencing homelessness make sense and cope with trauma. Policy and prevention should prioritise early intervention to reduce the mental health burden of trauma and homelessness. Additionally, creating support that empowers and builds resilience will encourage more positive management strategies.

## Background

Trauma and homelessness are closely related (Deck & Platt, 2015). Trauma is the psychological response to an event or experience that is out of the ordinary and has lasting impacts on all aspects of wellbeing, particularly mental health (Substance Abuse and Mental Health Services Administration [SAMHSA], 2014). Evidence suggests that there are two types of trauma (Sweeney et al., 2016; Wilkinson et al., 2017). Type 1 trauma focuses on a single event, whereas type 2 takes place when an event extends across a period of time. When people are exposed to multiple traumatic events taking place as single events and repetitively over time, they face complex or compound trauma. This is something that is common among people experiencing homelessness (Maguire et al., 2009).

Trauma is interlinked with homelessness in three ways (FEANTSA, 2017). First, prior experience of trauma is associated with homelessness. A recent systematic review (including 29 studies) found that nearly 90% of people experiencing homelessness had faced at least one adverse childhood event in their lifetime and 54% had faced four or more (Liu et al., 2021). Second, while someone is homeless, they are more likely to be the victim of trauma. Across the life-course, trauma becomes almost universal for people experiencing homelessness, with rates highest among women (Buhrich et al., 2000). A recent study looking at prevalence of trauma among people experiencing homelessness in a housing service in England found that homelessness amplified experiences of trauma and a third of respondents felt trauma hindered their ability to transition out of homelessness (Irving & Harding, 2022). Finally, becoming homeless and the experience of losing one’s home and the sense of loss and security has been associated with trauma, particularly psychological trauma (Goodman et al., 1991).

Experiencing and witnessing traumatic events singularly or repeatedly greatly affect people experiencing homelessness (Duncan et al., 2019), through shaping not only the way people understand their own needs and behaviors, but also how they relate to others and the wider society (Guarino & Bassuk, 2010). Although much of the previous evidence on trauma and homelessness has focused on quantifying the issue (Buhrich et al., 2000; Deck & Platt, 2015; Liu et al., 2021; Meinbresse et al., 2014), there has been growing evidence of qualitative literature exploring the nuances of experiences of trauma during homelessness.

Mental ill-health and substance use are also both a cause and consequence of homelessness (McNaughton, 2008; Padgett, 2020). The interconnection between mental health, substance use, and homelessness becomes more complex when considering the role of trauma. Losing one’s home, circumstances while in temporary living situations, and abuse before or after becoming homeless can lead to or exacerbate psychological trauma (Goodman et al., 1991). The high prevalence and recurrent nature of trauma during homelessness leads to heightened risk of post-traumatic stress and other related mental health challenges (Kim & Ford, 2006). The mental health impacts of trauma worsen for people the longer they are homeless (Wiewel & Hernandez, 2022). To cope with the impacts of current and previous experiences of trauma, people experiencing homelessness can turn to drugs or alcohol (McNaughton, 2008; Padgett, 2020). Much of the evidence exploring mental health, trauma, and homelessness, focuses on adverse childhood events (Koh & Montgomery, 2021; Liu et al., 2021) or is quantitative in nature (Buhrich et al., 2000; Deck & Platt, 2015; Kim & Ford, 2006; Liu et al., 2021; Meinbresse et al., 2014; Wiewel & Hernandez, 2022).

### Need for current systematic review

As part of effectively addressing homelessness, there is a critical need to understand and address trauma (Wiewel & Hernandez, 2022), particularly considering the role that trauma has in shaping responses to subsequent experiences and access to support/services (Harris & Fallot, 2001). Qualitative research exploring the impact of trauma during homelessness on mental health can provide a deeper, person-centered understanding of what it is like for people experiencing homelessness, how it impacts them, and how they cope with it. To date, no qualitative systematic review exploring trauma during adult homelessness and the mental health impacts has been published. Synthesizing this understanding provides the evidence on the contextual and complicated nature of trauma and its dialectical relationship as experienced by people facing homelessness. This synthesis of evidence would help inform development of homeless health practice and policy, as well as the implementation of trauma-informed care in homeless settings.

### Aim

This review aims to synthesize evidence from qualitative studies to explore the lived experience of trauma during homelessness and its impact on mental health (including substance use) from the perspective of adults (over 18 years of age) experiencing homelessness.

## Method

The review protocol was registered with PROSPERO (CRD42022349742). ASSIA, CINAHL, Cochrane, EMBASE, MEDLINE, Proquest theses and dissertations, PsycINFO, Scopus, and Web of Science were searched from inception until February 2024. Key words were developed relating to homelessness, trauma, mental health and substance use, and qualitative research. With the support of a librarian, these were mapped against relevant controlled vocabulary (e.g., MeSH, CINAHL headings) or key words, with truncations, exploding, and adjacency features used. Full database searches can be found in the Supplemental Material. No language, date, or geographical limits were applied. Gray literature searches were conducted using Google and selected charity and health and social care websites such as Crisis UK, Pathway, Homeless Hub, European Observatory on Homelessness. Forward and backward citation search of the included studies was also conducted.

### Co-Production Process

Using the Authors and Consumers Together Impacting on eVidencE (ACTIVE) framework as the basis for involvement across the review process (Pollock et al., 2019), this review was co-produced with people who have lived experience of homelessness. A strength of the ACTIVE framework for co-production of reviews is the transparency of who was involved, how they were recruited and involved, and what level their involvement was across the different processes. There are five levels of involvement from most to least involved: leading, controlling, influencing, contributing, and receiving. Table 1 outlines the 12 stages where involvement can take place as recommended by the ACTIVE framework and presents the level of involvement and examples of involvement undertaken in this specific systematic review.

**Table 1.** Involvement Within This Systematic Review Based on the ACTIVE Framework.

Review Process	Level of Involvement	Examples of Involvement
Develop questions	Influencing	With the researcher defined the inclusion criteria and definitions used for homelessness, trauma, and mental health.
Plan methods	Influencing	With the researcher identified, what involvement would look like at each of the stages and provided feedback on training requirements.
Write and publish protocol	Influencing	Reviewed the protocol and provided feedback and suggestions for change. Named as co-authors on protocol.
Develop search	Influencing	Reviewed the keywords and terms for the searches and provided suggestions for additional terminology or keywords.
Run search	Receiving	Received an explanation on the process used for running the search strategy over an online meeting.
Select studies	Controlling	After receiving training, they completed second screening for just over 20% of titles and abstracts, and over 30% of the full texts.
Collect data	Influencing	Reviewed the papers and identified key elements from the results sections of the paper.
Assess risk of bias	Receiving	Provided an update on the quality appraisal process.
Analyze data	Controlling	Undertook coding of a portion of the excerpts from the papers.
Interpret findings	Controlling	Assisted with identifying the preliminary themes and refining the themes.
Write and publish review	Influencing	Providing feedback into the implications for policy and practice for the study and reviewing and providing feedback on the draft of the paper.
Knowledge translation and impact	Controlling	Co-presenting at conferences. Co-authoring publications and abstract submissions.

*Note*. ACTIVE = Authors and Consumers Together Impacting on eVidencE.

Recruitment took place through inviting individuals through the existing network of the lead researcher and sending a general expression of interest request through a regional (public and practice involvement group focusing on public health) mailing list. This led to one man (JP) and three women (VB, JK, MO) coming forward. An individual with experience of providing services for this population and who works for a charitable organization (SK) was invited based on previously expressed interest in the topic. A partnership approach was used to seek continuous involvement from these individuals and ensure people were active members of the research team. Three bespoke training sessions were offered over the course of the review to support meaningful involvement: (a) overview of systematic review and what goes into a protocol and search strategy, (b) developing and operationalizing inclusion and exclusion criteria for screening and how to use Rayyaan (Ouzzani et al., 2016), and (c) how to code and analyze data.

### Eligibility Criteria

Using Rayyan, two reviewers independently screened all titles and abstracts against pre-determined inclusion and exclusion criteria, followed by full-text screening for any potentially eligible study. At abstract and full-text screening discussions, to reach consensus on any discrepancies, the two reviewers met to discuss conflicting opinions. In most cases, this led to a consensus. However, where a consensus could not be reached the senior author reviewed the abstract/paper as a third independent reviewer and made the decision on inclusion. The lead and senior authors reviewed the final list of included papers following data extraction to confirm the final papers included in the synthesis. English versions of non-English papers were identified, and where these were not found, papers were translated by someone who spoke the language or through Google translate. Relevant data were extracted (e.g., methodology, sample characteristics, homelessness, and findings) by the lead author and checked by another co-author. Data extracted from the results/finding sections of the included papers were restricted to direct quotes reflecting on experiences of trauma during periods of homelessness and its subsequent impact on mental health. Additionally, the immediate contextual information preceding or following the quotation was also extracted to support with contextualizing quotes as part of the analysis for this review. When information was missing or full texts could not be obtained, corresponding authors were contacted.

Any qualitative research reporting lived experiences of trauma and its impact on mental health during homelessness in adults was eligible. Where studies had populations inclusive of 16 to 24, if data from those 18 and over could be easily identified, they were still included. Based on discussion with people in practice and people with lived experience, a decision was made to exclude studies focusing on entire communities experiencing homelessness (such as refugee and asylum-seeking populations). This was due to the assumption that these populations likely face heightened experiences of trauma that are unique to their specific circumstances.

To reflect the range of impacts trauma could have, a broad definition of mental health was applied. This could include: mental wellbeing, resilience and other positive mental wellbeing outcomes, substance use, common mental health disorders (such as anxiety, depression), and more severe mental health disorders (such as schizophrenia and other psychotic disorders). Trauma was operationalized using the SAMHSA definition:

Individual trauma results from an event, series of events, or set of circumstances that is experienced by an individual as physically or emotionally harmful or life threatening and that has lasting adverse effects on the individual’s functioning and mental, physical, social, emotional, or spiritual well-being. (SAMHSA, 2014)

This definition was used as it aligned with the preliminary model used to inform the best-fit framework synthesis (described below) and acknowledges the subjective nature of trauma and identifying traumatic events. Furthermore, it provided a holistic representation of the range of events and experiences of trauma that would be reflected in qualitative literature.

Studies were excluded if they did not focus on homelessness or focused solely on experiences of trauma and did not explore its impact on mental health or vice versa. Additionally, as the study was focused on trauma during periods of homelessness, where there was ambiguity on whether trauma took place during such periods, studies were excluded. This led to several studies being excluded from both peer-reviewed sources and particularly gray literature sources.

### Quality Assessment

Quality assessment used a two-stage process adapted from (Britten & Pope, 2011). First, the Critical Appraisal Skills Programme (CASP) Qualitative Studies Checklist was used to evaluate overall clarity, rigor, and appropriateness. Quality was not used as an exclusion criterion. Instead, a modified rating scale based on Dixon-Woods et al. (2007) and used by Malpass et al. (2009) and Muir et al. (2023) was used to reflect the overall quality as well as the relevancy of the studies to the overall review aim. Studies were rated as (A) a key paper that was most relevant and conceptually rich, with no or few quality issues; (B) a secondary key paper, that was relevant but with limited themes and data, and/or some quality issues; or (C) satisfactory, that was less relevant to the review and/or had major quality issues. Led by the lead author, data extraction was completed simultaneously with the quality appraisal and checked by a second author. Discrepancies were resolved through discussion or where needed by involving a third author.

### Data Synthesis

A “best-fit” framework synthesis was undertaken, which follows seven key steps (Booth & Carroll, 2015; Carroll et al., 2013). First, the review question was formulated. Two separate activities took place as part of the second step. While the databases were searched, relevant frameworks, models, or theories were identified. At this stage, the SAMHSA concept of trauma (three Es) was identified (SAMHSA, 2014). This framework conceptualized trauma into three components: event(s), experiences of event(s), and effect. Events or circumstances represent the actual situation or threat, and reflects the potential for it to have taken place as a one-off or repetitively. The way someone experiences the event determines whether something is traumatic and recognizes the personalized nature of the trauma. Finally, the effect focuses on the long-lasting adverse consequences, which can take place immediately following an event or can be delayed. Although the model is not specific to homelessness, this model was chosen for its inclusive approach to trauma, and prominence in trauma research and practice. Step 3 involved simultaneously extracting the data and completing the quality appraisal while also identifying the a priori coding framework. In this case, it involved using *Homeless Events*, *Experiences*, and *Effects (broken down into short, medium, and long-term*). In step 4, all data were coded against this a priori framework in NVivo (QSR International Pty Ltd, 2018). For people with lived experience, they undertook coding of a portion of the results using MS Word. Step 5 involved secondary inductive coding before moving onto step 6 where the a priori framework and the new codes are amalgamated into an expanded coding framework. Finally, step 6 involves exploring the relationship between themes or concepts and creating a new model (as illustrated in Findings section).

## Findings

### Description of Studies

**Figure 1.**
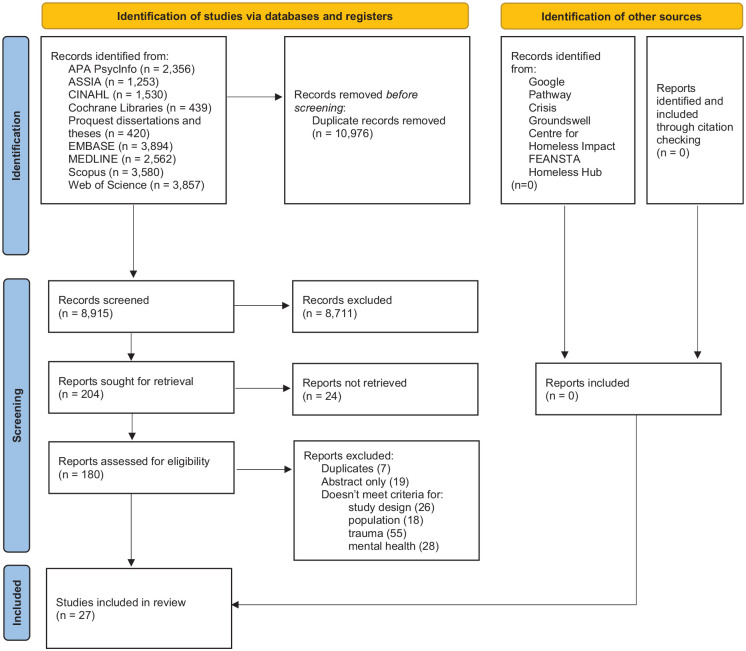
PRISMA flowchart of included studies.

Twenty-seven studies were included (see Figure 1 for PRISMA flowchart) in the analysis. No further studies were identified through gray-literature searches; however, two of the included studies were dissertations. Further descriptive summary characteristics of included studies are in Table 2. The analysis included a total of 909 adults experiencing homelessness, aged 18 to 70 years of age. Fourteen studies explored specific subgroups of populations, including severe mental illness (Bonugli et al., 2013; Narendorf, 2017), Indigenous (N. E. Elliott, 2018), veterans (Keene, 2013), substance use/drug use (Kim & Roberts, 2004; Neale, 2001; Nettleton et al., 2012; Nicholls & Urada, 2021), mothers/guardians (Kirkman et al., 2015) without children (Osuji & Hirst, 2021), pregnant while homeless (Sadeghi et al., 2021), separation of family in childhood/adolescent (Shaikh & Rawal, 2019), attempted suicide (Silva de Castro et al., 2019), and older women (Sutherland et al., 2022). Definitions of homelessness ranged from no specific definition to more specific definitions included time spans and specific forms of homelessness (e.g., street sleeping, shelter use). Studies recruited samples from the United States (*n* = 9), Canada (*n* = 5), Australia (*n* = 4), United Kingdom (*n* = 4), Brazil (*n* = 3), Ethiopia (*n* = 1), and Iran (*n* = 1), and mostly reported participants from urban settings.

**Table 2.** Brief Descriptive Summaries of the 27 Included Studies With Quality Appraisal (Key Paper: A/B; Satisfactory Paper: C).

First Author (Year); Country; Publication Type	Aim and objectives	Homeless Sample Size (Female), Ages	Homelessness	Data Collection, Recruitment, Analysis	Quality Appraisal
Biscotto (2016); Brazil; Journal article	To understand the life experience of homeless women	*N* = 10(10F), 22–44	Street sleeping and sporadic shelter use Women	Interviews, through a shelter, social phenomenology	B
Bonugli (2013); United States; Journal article	To increase understanding of victimization among homeless women living with severe mental illness as perceived by those who have lived the experience	*N* = 15(15F), 22–62	Shelter use Experiencing severe mental illness Women	In-depth semi-structured interviews, purposive sample through a shelter, content analysis	A
Dickins (2023); United States; Journal article	To develop understanding of the determinants of trauma, risk, exposure, and outcomes among women experiencing homelessness	*N* = 10(10F), range not reported (18 and older)	Currently homeless Health Resources Services Administration definition Women	In-depth semi-structured interviews (part of larger study), targeted stratified recruitment through a homeless organization, deductive-inductive content analysis	C
Elliot (2018); Canada; Dissertation	To understand what are the intersections of trauma, mental health, and traditional knowledge for urban indigenous homeless people?	*N* = 16(5F), range not reported (18 and older)	Street involved or wider homelessness Indigenous	Interviews, through a community outreach team, analysis informed by Indigenous inquiry framework	A
Haile (2020); Ethiopia; Journal article	To explore the factors associated with onset of substance use and its continued use, patterns of substance use and its social and health consequences	*N* = 15(15F), age not reported	Those sleeping in designated public spaces or shelters, or those who are “roofless” Female	In-depth interviews, purposive sample from a shelter, no specific analysis method named	C
Huey (2012); United States; Journal article	To understand how do existing mental health services respond to women’s psychological trauma	*N* = 79(79F), 18–70	Currently homeless (not defined) Women	In-depth semi-structured interviews (part of a larger study), nonprobability sample through services (self-selecting), thematically (no explicit method)	C
Keene (2013); United States; Dissertation	To explore the meaning of homelessness to female homeless veterans, the risk factors for homelessness and services necessary to help exit the homeless cycle	*N* = 6(6F), 41–60	Without a roof over one’s head, living in a shelter or transitional housing, with a friend or family, in the domiciliary homeless program or in an automobile Veterans Women	Semi-structured interviews, purposive sample recruited through homeless veteran, veteran, and homeless services, similar to descriptive phenomenology	A
Kim (2004); United States; Journal article	To examine the contexts from which homeless men have emerged; the environments in which the homeless men currently live; and define the details of and connection between the whole culture and the particulars of the men’s lives that have brought them to the place from which they are currently speaking	*N* = 10(0F), range not reported (18 and older)	Current or past history of homelessness (not defined) Substance use Men	In-depth interviews, through a substance use provider staff, no specific analysis method named	C
Kirkman (2015); Australia; Journal article	To report on women’s experiences of being homeless with their children	*N* = 12(12F), range not reported	Inclusive definition of homelessness Mothers or guardians	Semi-structured interviews, through services, thematic analysis informed by theory of the narrative mode of thought	B
Lewinson (2014); United States; Journal article	To explore types of trauma and adversity prior to and during housing at budget hotels.	*N* = 21(21F), 19–64	Lived exclusively in a budget hotel for at least 2 weeks as a result of housing displacement Women	Semi-structured interviews, convenience and snowball sampling through a budget hotel, narrative and categorical content analysis	B
Li (2020); United States; Journal article	To investigate vulnerabilities for women; identify existing barriers for women; explore the relationship between mental health and homelessness for women	*N* = 32(17F), range not reported	Living on the streets, shelter, staying with a friend, recently in rental	In-depth interviews, convenience through library, grounded theory, and thematic analysis	C
McNaughton (2008); United Kingdom; Journal article	To longitudinally understand transitions through homelessness	*N* = 28(13F), 25–60	Repeated episodes of homelessness; street sleeping, lack of permanent accommodation, moving between hostels and bed and breakfasts	Longitudinal interviews over a year, recruited through a provider, narrative matrix analysis	C
Morrell-Bellai (2000); Canada; Journal article	To identify how people become homeless and why some individuals remain homeless for an extended period of time or cycle in and out of homelessness (the chronically homeless)	*N* = 29(9F), 18–61	Shelter users and shelter avoiders	In-depth semi-structured interviews (part of a larger study), through shelters and selected from larger study, group qualitative analysis	B
Narendorf (2017); United States; Journal article	To examine the intersection of homelessness and mental health	*N* = 26(5F), 18–25	Self-identified within the last 12 months, or reported currently living in a shelter on or the streets Severe mental illness	Semi-structured interviews (part of a larger study), through a publicly funded voluntary, short-term crisis stabilization unit for uninsured patients, grounded theory	C
Neale (2001); United Kingdom; Journal article	To offer a detailed analysis of drug users’ own accounts of being without a home	*N* = 200(69F), 15–47	Sleeping rough, living in emergency accommodation, or staying in “care of others” out of necessity rather than through choice Drug users	Semi-structured interviews (part of larger study), recruited through hospital and drug agencies/pharmacies, framework analysis	B
Nettleton (2012); United Kingdom; Journal article	To present an analysis of sleeping practices amongst homeless drug users who make use of emergency hostels and night shelters	*N* = 40(11F), 21–54	Emergency hostels and night shelters Drug users	Interviews, maximum variation sampling from a range of services, thematic analysis	C
Nicholls (2021); United States; Journal article	To explore the intersection of homelessness and substance use-related problems and its compounded impact on barriers for recovery, along with facilitators to treatment and recovery	*N* = 22(9F), 22–63	Unstably housed or homeless Actively using drugs	In-depth interviews and focus group discussions (part of larger study), purposive homogenous sampling strategy through library; iterative thematic analysis	C
Osuji (2021); Canada; Journal article	To explore the lived experiences of women without children experiencing housing instability and homelessness	*N* = 12(12F), 34–65	Housing instability and homelessness Without children Women	Unstructured interviews and observations, purposeful, convenience and snowball sampling through homeless service, interpretative analysis (hermeneutics), and thematic analysis.	C
Phipps (2021); Australia; Journal article	To examine the experiential perspectives of women becoming and experiencing homelessness	*N* = 11(11F), 30–66	Previously having been homeless and currently housed in stable accommodation Women	Auto-driven photo-elicitation and in-depth interviews, recruitment through services, thematic analysis	B
Piat (2015); Canada; Journal article	To examine how homeless individuals with mental illness experience pathways into homelessness.	*N* = 219(79F), range not reported (18 and older)	Absolute homelessness, precariously housed (extensive definitions provided) Mental disorder with or without co-occurring substance use (DSM-IV criteria)	Narrative interviews, randomly selected from larger sample recruited through various services (purposeful adjustments to ensure diverse cross-section), constant comparative analysis and grounded theory	C
Rosa (2015); Brazil; Journal article	To bring out reflections regarding situations of violence in the lives of women who were living on the streets	*N* = 22(22F), range not reported (18 and older)	Living on the streets Women	Cartographic method including ethnography with interviews, recruitment through a provider/center, Foucauldian analysis of discourse	B
Sadeghi (2021); Iran; Journal article	To understand how homeless pregnant women experience homelessness and pregnancy	*N* = 13(13F), 20–50	No definition for homelessness Experience pregnancy while homeless Women	Semi-structured interviews, snowball sampling through an urban treatment center, thematic analysis	B
Shaikh (2019); Canada; Journal article	To explore the personal experiences of participants with homelessness, family separation, and mental health and addiction issues	*N* = 13(3F), 18–57	Lifetime homelessness (inclusive definition) Separation from family in childhood/adolescence Mental health or substance abuse issue	Semi-structured interviews, criterion and snowball sampling through various providers, circular, and interactive process	C
Silva de Castro (2019); Brazil; Journal article	To understand the vulnerabilities of street adults to suicidal behavior	*N* = 8(2F), 22–57	Living in the street and registered in the social approach sector of the Specialized Referral Center for Social Assistance (CREAS). Attempted suicide	Interviews, no recruitment details provided, content analysis	C
Sutherland (2022); Australia; Journal article	To explore the healthcare needs and barriers to health services in older homeless women	*N* = 22(22F), 48–82	Living in short-term transitional housing, emergency accommodation, some rough sleeping Older women	Questionnaire and semi-structured interviews, recruitment through homeless organizations, thematic analysis	B
Thomas (2021); Australia; Journal article	To explore the qualitative dimensions of stigma related to substance use for women experiencing homelessness	*N* = 10 10F), 19–mid 60s	Chamberlain and MacKenzie’s (1992) typology of homelessness (primary, secondary, and tertiary). Women	Ethnography participant observation with life history interviews, through public spaces and service providers, thematic and framework analysis	C
Williams (2011); United Kingdom; Journal article	To examine how people talk about their experiences of homelessness	*N* = 8(1F), 24–64	Self-identified as homeless	Narrative interviews, recruited through homeless shelters, vignettes, and coding	C

### Themes

**Figure 2.**
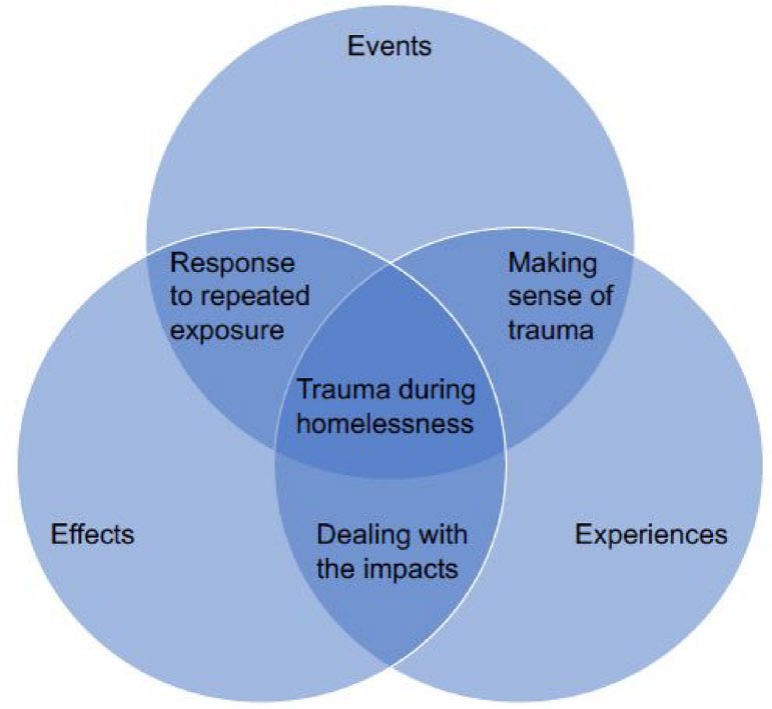
Conceptual model for trauma during homelessness and the mental health impacts.

Each theme has subthemes (a table documenting which studies are related to each theme and subtheme can be found in Supplemental Material). The themes illustrated the interconnectivity between events, experiences, and effects (three Es model) when considering how trauma shapes the lives of people experiencing homelessness, and its cyclical nature. Figure 2 begins to conceptualize a model for trauma during homelessness and its mental health impacts, with the themes from this review informing the overlays between the original model.

#### Making Sense of Homelessness as a Trauma

Across the studies, homelessness was linked with psychological trauma, expressed in terms of shock, isolation and detachment, and wider accommodation-based issues (Biscotto et al., 2016; Bonugli et al., 2013; N. E. Elliott, 2018; Haile et al., 2020; Huey et al., 2012; Keene, 2013; Kirkman et al., 2015; Lewinson et al., 2014; Narendorf, 2017; Neale, 2001; Osuji & Hirst, 2021; Phipps et al., 2021; Shaikh & Rawal, 2019; Sutherland et al., 2022). These experiences of traumatic events often highlighted the absence of choice for people experiencing homelessness. The way events during homelessness were experienced is what differentiates negative experiences from trauma; this theme is reflected in the new model through the intersection between events and experiences.

##### Experience of Homelessness

Words like “ashamed,” “worthless,” “lost,” “embarrassed,” “helplessness,” “unwanted,” “hopeless,” and “failure” were often used to describe present situations of homelessness (Biscotto et al., 2016; E. Elliott et al., 2002; Haile et al., 2020; Keene, 2013; Kim & Roberts, 2004; Kirkman et al., 2015; Morrell-Bellai et al., 2000; Osuji & Hirst, 2021; Phipps et al., 2021; Sutherland et al., 2022), and highlighted the preconceptions of what it meant to be homelessness. These emotions and feelings became the context shaping understanding of experiences. In some cases, this translated into feeling isolated or detached (Haile et al., 2020; Huey et al., 2012; Osuji & Hirst, 2021; Shaikh & Rawal, 2019), in others this led to states of shock (Huey et al., 2012; Keene, 2013; Narendorf, 2017). One woman described “even if I had fifty people around me, I still felt alone, because I was so detached from everything else and everyone [. . .]” (Osuji & Hirst, 2021). For those in shock, the confusion and disbelief took many forms; one person shared “I can’t believe that I’m homeless” (Huey et al., 2012), where as another person described their current situation as a “dark downward spiral” (Keene, 2013). Questioning how one became homeless was common across studies. The traumatic nature of homelessness was highlighted by one woman who had escaped domestic violence “. . .And you know, if I knew it was this bad, I don’t know what I would have done—probably still be married” (Lewinson et al., 2014).

##### Chaos Surrounding Homelessness

The wider circumstances surrounding homelessness exacerbated experiences of trauma (Bonugli et al., 2013; N. E. Elliott, 2018; Keene, 2013; Kirkman et al., 2015; Lewinson et al., 2014; Narendorf, 2017; Neale, 2001; Piat et al., 2015; Rosa & Brêtas, 2015; Thomas & Menih, 2021). Often stories highlighted that the chaos surrounding housing situations led to trauma, from the accommodation environment to its adequacy and appropriateness. This was highlighted by one situation:

Some places you go to, it sucks more than being homeless. Not even the shelter; I never go to shelters, hell with that, that’s worse than hell. I’d rather be outside because you are free. I am not stuck in some of those bug infested, mouldy places and stuff like that. Some of the places you rent, it’s worse than hell. I’d rather sleep in fresh air and freeze to death. (N. E. Elliott, 2018)

A woman accommodated with their son explained “we couldn’t even come out of our room to go to the toilet,” because of the violent environment they were accommodated in (Kirkman et al., 2015). Apart from accommodation challenges, a few studies highlighted needs around caring responsibilities, criminal justice involvement, accessibility requirements, and health concerns (Keene, 2013; Kirkman et al., 2015; Narendorf, 2017). Rarely was homelessness experienced without surrounding complexities, which added to experiences of trauma.

#### Dealing With the Mental Health Impacts of Trauma

The most universal theme across studies was how trauma impacted the mental health of people experiencing homelessness (Biscotto et al., 2016; Bonugli et al., 2013; Dickins et al., 2023; N. E. Elliott, 2018; Haile et al., 2020; Huey et al., 2012; Keene, 2013; Kim & Roberts, 2004; Kirkman et al., 2015; Lewinson et al., 2014; Li & Urada, 2020; McNaughton, 2008; Morrell-Bellai et al., 2000; Narendorf, 2017; Neale, 2001; Nettleton et al., 2012; Nicholls & Urada, 2021; Osuji & Hirst, 2021; Phipps et al., 2021; Piat et al., 2015; Rosa & Brêtas, 2015; Sadeghi et al., 2021; Shaikh & Rawal, 2019; Silva de Castro et al., 2019; Sutherland et al., 2022; Thomas & Menih, 2021; Williams & Stickley, 2011). This captures the intersection between the experiences and effects within the three Es model. As one woman described “Bruises heal. The mental stuff doesn’t” (Sutherland et al., 2022). Although the impacts varied, the recognition that trauma shaped people’s mental health, wellbeing, and substance use was very prominent in the evidence.

##### A Constant State of Fear

A common impact of trauma while homeless was an ongoing state of fear whether because of violence, crime, uncertainty, or personal safety (Biscotto et al., 2016; Keene, 2013; Kim & Roberts, 2004; Li & Urada, 2020; Morrell-Bellai et al., 2000; Narendorf, 2017; Nettleton et al., 2012; Piat et al., 2015; Rosa & Brêtas, 2015; Sadeghi et al., 2021; Shaikh & Rawal, 2019; Sutherland et al., 2022; Thomas & Menih, 2021). In some studies, fear was a response to both experiencing and witnessing sexual assault and rape (Kim & Roberts, 2004; Li & Urada, 2020; Narendorf, 2017; Piat et al., 2015; Rosa & Brêtas, 2015; Sadeghi et al., 2021), which was perpetuated by the extreme circumstances surrounding traumatic experiences. After being raped while unconscious, one woman sleeping on the streets explained she woke up “terrified” and underwent “shakes” (Li & Urada, 2020). By contrast, another woman reported how overhearing assaults led them to leave their shelter and return to the streets out of fear: “I just left whatever I had there and I never went back. . .cause I was too scared” (Piat et al., 2015). Fear of violence was often something that dominated the minds of people experiencing homelessness after they either experienced or witnessed extreme violence (Biscotto et al., 2016; Rosa & Brêtas, 2015; Sadeghi et al., 2021; Shaikh & Rawal, 2019; Thomas & Menih, 2021). As one man explained he was constantly worrying about “when am I going to get robbed, when am I going to get stabbed or who is gonna try to punch me out?” (Shaikh & Rawal, 2019). This constant worrying was not unique to one experience, and led to those on the streets often “. . .stay[ing] awake the whole night out of fear” (Rosa & Brêtas, 2015).

##### Anxiety and Depression

Anxiety was a consequence of trauma in some cases (Bonugli et al., 2013; Huey et al., 2012; Keene, 2013; Narendorf, 2017). This involved being extremely overwhelmed by situations as well as having panic attacks. A man who was sleeping on the streets explained:

I’m out in the cold. And the burden hit me hard, like, man it took all the breath from me when that happened, it just, my chest got real heavy, my mind started racing and wandering trying to figure out, man, what I’m going to do. . .. (Narendorf, 2017)

A woman who was a survivor of domestic violence and then became homeless found the whole experience so overwhelming that when “It got really bad. I just wanted to run in front of a truck or something” (Huey et al., 2012). Longer-term, depression, and suicidal ideation were commonly reported as consequences to experiences of trauma (Biscotto et al., 2016; Bonugli et al., 2013; N. E. Elliott, 2018; Huey et al., 2012; Kim & Roberts, 2004; Li & Urada, 2020; Morrell-Bellai et al., 2000; Narendorf, 2017; Neale, 2001; Phipps et al., 2021; Silva de Castro et al., 2019; Sutherland et al., 2022; Thomas & Menih, 2021). One male living on the streets explained:

[. . .]Yeah, wow, sadness! I thought I was not going to get anything, nothing was going on for me, nothing was going to work. This is what forms the idea [. . .]. With knife, razor, screwdriver, bleach, with soap powder. (Silva de Castro et al., 2019)

##### Substance Use as a Management Strategy

People across the studies commonly discussed using drugs or alcohol to “cope” with homelessness and trauma (Biscotto et al., 2016; Bonugli et al., 2013; Dickins et al., 2023; N. E. Elliott, 2018; Haile et al., 2020; Huey et al., 2012; Kim & Roberts, 2004; McNaughton, 2008; Neale, 2001; Nettleton et al., 2012; Nicholls & Urada, 2021; Osuji & Hirst, 2021; Shaikh & Rawal, 2019; Thomas & Menih, 2021; Williams & Stickley, 2011). Substance use was often seen as a way to deal with memories and experiences. For many, there was a belief that substances helped “. . .kinda makes it easier to deal with. . .” (Nicholls & Urada, 2021), or that they “. . .drink to forget myself (forget the miseries of life); the alcohol makes me relax” (Haile et al., 2020). Substances were often seen quite positively and frequently as the normal solution to cope; one man explained “it’s just the norm. You begin to think you’re just doing a normal thing” (McNaughton, 2008) and another explaining “Alcohol was always the answer” (Kim & Roberts, 2004).The use of substances as a form of escapism was common across the studies, illustrated through the following:

You need the smack. . . I’ve been in some state, but the smack kind of kept me sane. It made me able to handle my homelessness and that. . . It was the smack that got me through it. It deadened my thoughts and just kind of froze me. (Neale, 2001)

##### Strategies to Feel Safe

After experiencing trauma, there was a natural response to put strategies in place to maintain a sense of safety. The specific reason and response was different for each person, but the overall concept appeared across several studies (Biscotto et al., 2016; Bonugli et al., 2013; N. E. Elliott, 2018; Keene, 2013; Kirkman et al., 2015; Lewinson et al., 2014; Nettleton et al., 2012; Rosa & Brêtas, 2015; Sadeghi et al., 2021). For people who had been sleeping on the streets when they experienced trauma, the strategy often involved going to a shelter (Biscotto et al., 2016; N. E. Elliott, 2018; Nettleton et al., 2012; Rosa & Brêtas, 2015). Despite shelters not necessarily being perfect, one person explained it was better than the alternative:

But at the end of the day, it was a roof over my head. It was better than where I was sleeping, so I didn’t give a shit that it was a dirty kitchen. . . It was like I had fallen into heaven, do you know what I mean? I wasn’t laying on a freezing cold park bench, worrying that someone was going to cut my throat when I was asleep, or try to jack me while I was asleep, you know. I mean I’ve been stabbed and that. I’ve been kidnapped, you know what I mean. (Nettleton et al., 2012)

For those already in hostels, the response was often minimizing contact with others in the accommodation (Kirkman et al., 2015). Others who were afraid or apprehensive about hostels often resorted to finding ways to make sleeping in a car their sanctuary:

I went to the place where I’ve had a storage unit and I knew that place locked their gates at 9. So, I secured the back of the car and put pillows for the kids to sleep. I pretty much stayed up all night watching them (cries). (Lewinson et al., 2014)

##### Responses to Repeated Exposure to Trauma

Unsurprisingly people who experienced prolonged homelessness faced trauma more than once, in different forms, and often across their life. In many situations, people shared stories illustrating secondary trauma (e.g., death of friends or witnessing violence toward others) in addition to primary trauma. This was illustrated by a two-spirited Indigenous man who explained “. . .I’ve bled, I have been cut, I’ve been shot, I’ve been knifed. . .I have had friends executed in front of me” (N. E. Elliott, 2018). This led to longer-term responses that led to people either accepting their situation or realizing that things could get better (Bonugli et al., 2013; Dickins et al., 2023; N. E. Elliott, 2018; Li & Urada, 2020; McNaughton, 2008; Neale, 2001; Osuji & Hirst, 2021; Phipps et al., 2021; Rosa & Brêtas, 2015). The cyclical nature of trauma and particularly the role long-lasting adverse effects have in shaping understanding of future traumatic events is reflected in our new model as the overlap between effects and events.

##### Acceptance and Resignation as a Response

The constant trauma faced, and often secondary trauma experienced led to several people reaching a point where they accepted or resigned to acknowledging trauma as a norm. Phrasing such as “given up on everything” (Li & Urada, 2020), “just go with it” (N. E. Elliott, 2018), “what’s the point” (Neale, 2001), were often used when describing their present circumstances. The concept of survival was present in several studies as a response to repeated trauma (Biscotto et al., 2016; Bonugli et al., 2013; Dickins et al., 2023; N. E. Elliott, 2018; Kim & Roberts, 2004; McNaughton, 2008; Osuji & Hirst, 2021), whereby people described “. . . you just try to survive by however means. . .” (Keene, 2013). Often experiences that people would have otherwise found to be negative or traumatic, were negated given their current approach to life. Acceptance of the current situation was also common when considering death. Given the amount of death people had witnessed while homeless, this sometimes led to a bleak outlook that their own death was inevitable when they were homeless. This was illustrated by one woman who had been on the streets for 2 years and said: “I wasn’t even supposed to be here today at this table recounting my life” (Rosa & Brêtas, 2015). The perspective that death was unavoidable with longer-term experiences of homelessness was common among studies.

##### Hope for a Better Tomorrow as a Response

In a minority of studies, there were stories sharing a slightly more positive approach to current circumstances despite all the trauma people had faced (N. E. Elliott, 2018; Phipps et al., 2021). This came through as hope, resilience, and optimism for a better future for themselves that carried people through their current situation. In one experience, an Indigenous man living in a city worked to make a difference for themselves and others, shared: “I really got tired of going to the memorials and everything you know, so I just started being support for everybody else” (N. E. Elliott, 2018). In a study of women experiencing homelessness, there were several stories focused on a sense of hope that things would be better, where one woman said: “I think it was definitely hope, so believing that there is something else and that it cannot end like this” (Phipps et al., 2021).

Although less common across all the studies, the potential of a positive outlook as a protective factor while someone experiences homelessness was important for longer-term impacts.

## Discussion

This systematic review of qualitative evidence focused on lived experiences of trauma during adulthood homelessness and the impact it had on mental health and substance use (see Table 3 for an overview). The experiences illustrated the detrimental impact homelessness has on mental wellbeing, particularly when homelessness in itself was a trauma, and people faced and witnessed trauma on multiple occasions. This is illustrated through the interconnectivity shown in the revised model presented. The emotional toll of becoming and being homeless cannot be ignored, as this was often the precedence for an interconnected cycle of disadvantage and hardship. Guarino and Bassuk (2010) argued that the negative impacts of trauma shape all aspects of functioning for people experiencing homelessness. An Australian study found that compared to those self-identifying as homeless, people who refused to self-identify as homeless had better wellbeing and mental health outcomes (Walter et al., 2015). Identifying as homeless led to people having to navigate preconceived notions and assumptions, which, as shown in our review, added to the trauma of becoming homeless. Services labeling people as homeless need to be aware of the negative impacts this might have for people and ensure that support is available to allow people to manage their emotions and feelings around becoming homeless.

**Table 3.** Critical Findings.

From a “best-fit” framework synthesis of 27 studies, covering 909 adults experiencing homelessness across seven countries, we identified three themes to understand the impact of trauma in the context of homelessness on mental health and substance use. These included making sense of homelessness as a trauma, dealing with the mental health impacts of trauma (including management strategies), and responses to repeated exposure to trauma.

Isolation and detachment were often consequences of becoming homelessness. This was consistent with existing research (Bower et al., 2018; Grigsby et al., 1990; Rokach, 2005; Sanders & Brown, 2015). A U.K. wide survey found that over 60% of people experiencing homelessness identified as lonely and over a third often felt isolated (Sanders & Brown, 2015). This isolation coupled with the shame and stigma from negative service encounters, made it harder for people to seek support (Sanders & Brown, 2015). Grigsby et al. (1990) suggested that becoming homeless was often linked with loss of social support, but it was at this initial stage that people were most likely to be receptive to support and had the least detrimental impact on mental health longer-term. However, the longer someone remained homeless, the more entrenched they became within homeless communities and the worse their mental wellbeing was. Building on the existing evidence quantifying the negative mental health impacts of trauma, our findings synthesize the rich narratives on lived experience of trauma during homelessness and provides deeper insights into its impact on mental health. Thus, policy and practice should prioritize interventions early on in experiences of homelessness to ensure that people are provided with appropriate housing and health and social care support to transition out of homelessness and prevent unnecessary deterioration of mental health. Table 4 presents a further summary of implications and recommendations that emerge from this review of evidence for practice and policy and for further research.

**Table 4.** Implications and Recommendations for Practice, Policy, and Research.

*Practitioners* are encouraged to provide early psychosocial support during experiences of homelessness to help with the initial shock and response. Practitioners are encouraged to consider the implications of labeling someone as homeless as depending on how someone feels about the label it could have detrimental impacts. Working with people with lived experience of homelessness has the potential to ensure support and service is provided in an appropriate and accessible way. Practitioners should consider how they can build resilience and empower people experiencing homelessness, so they begin to utilize positive management strategies. *Policies* should consider how homelessness is defined in national and local policies and carefully consider prescribing homeless labels as this can lead to unnecessary stigma and shame. Although policies should encourage early intervention to reduce long-term homelessness, there needs to be added attention to those who have been homeless for longer as they will likely have the most detrimental mental health impacts from trauma. *Researchers* should seek to better understand the causal pathways for trauma during homelessness and the impacts on mental health, with a particular focus on modifiable factors as an area for future intervention. Further research is needed in non-urban settings where evidence is limited.

Services and policy can play an important role in addressing trauma and its impact for people experiencing homelessness. Recently, there has been increasing evidence supporting implementation of trauma-informed approaches across homeless services (Bransford & Cole, 2019; Haigh et al., 2012; Hopper et al., 2010; Schneider et al., 2021). This review’s findings emphasize the importance of creating services that are trauma informed in order to mitigate the negative experiences with the way services can sometimes be delivered. Support needs to be provided in settings and approaches that are appropriate, accessible, and non-stigmatizing. Working with people with lived experience of homelessness could support with developing and adapting services to mitigate experiences of trauma when accessing support.

Mental health impacts from trauma ranged from fear early on to anxiety, depression, and suicidal ideation later on. Research has previously found heightened rates of anxiety and depression in people experiencing homelessness (Gutwinski et al., 2021; Hossain et al., 2020; Maestrelli et al., 2022). With trauma escalating these issues, our findings emphasize the importance of early intervention in preventing longer-term mental health impacts through providing stories illustrating the consequences of inaction. Specific support for anxiety and depression should consider the intersection of trauma and homelessness to ensure that all contributing factors are addressed. Our findings highlight that self-management approaches for dealing with negative mental health impacts can vary from positive approaches focused on increasing a sense of safety to maladaptive coping approaches such as the use of drugs and alcohol. Existing evidence has explored the reciprocal relationship between substance use, trauma, and homelessness (McNaughton, 2008; Padgett, 2020), but further research is needed to better understand the casual pathways or modifiable factors to support the different management approaches. With this evidence, policy and practice could be developed and tailored to empower people experiencing homelessness to use positive management strategies.

The cumulative nature of the mental health impacts from repeated exposure to trauma are important to consider. This review found a minority of experiences where resiliency and hope were the response to repeated exposure to traumatic events, rather than accepting or resigning oneself to the inevitability of trauma. This highlights the need for practice and policy to tailor available approaches to address the different responses to trauma. A negative outlook on future potential for change could lead to those experiencing homelessness disengaging from support services and wider society (Harris & Fallot, 2001), which in turn can result in chronic homelessness. Although there is a need to focus on preventing trauma, our findings suggest that it is imperative that trauma does not become a norm in everyday life for people experiencing homelessness. Future research should help identify ways to offer practical support and safety for people with no secure abode, as well as bolstering optimism that a journey out of homelessness is possible alongside trauma-informed care to support recovery. This evidence can subsequently support with developing and strengthening practices and policies for homelessness at local, national, and global levels.

### Strengths and Limitations

To the best of our knowledge, this is the first comprehensive systematic review to date exploring trauma experienced during homelessness in adulthood and the impact it has on mental health and substance use. Although this review drew on studies covering different countries, ethnicities, ages, and compositions of homelessness, much of this evidence was focused on urban areas. Further research is needed on less urbanized contexts such as rural or coastal communities. Additionally, none of the studies explicitly explored the relationship between trauma and mental health or substance use. Rather most of the included studies had a primary focus on homelessness where the relationality between trauma and mental health was a secondary component. We were limited by the quotes within the included studies, so this may not be inclusive of all experiences. Additionally, some studies may have not been published, which meant that we were unable to reflect on people’s experiences outside of published literature. Nevertheless, this review begins to highlight the ways in which trauma during homelessness shape trajectories of mental health and substance use—often with negative repercussions. A further strength of this study was its involvement of people with lived experience of homelessness as active members of the review team. Through using the ACTIVE framework (Pollock et al., 2019), we were able to plan and execute involvement across all stages of the review, thus not only improving the experience of those involved by developing their knowledge and skills, but also improving the quality, relevance, and impact of this review for the wider homelessness and health sector.

There are several limitations to this review. First, this review focused on trauma during homelessness in adulthood. However, determining whether participants were speaking about trauma during experiences of homelessness or trauma that took place earlier in their life was often a challenge. Not being able to ascertain the timepoint when trauma took place was often a reason for exclusion of studies in both peer-reviewed evidence and gray literature. Best efforts were made to use the entire context provided in the results to determine whether trauma was associated with homelessness, but identifying the timeline of experiences was often a challenge. One of the other issues that arose was due to the cumulative nature of impacts for trauma, which meant that we were unable to disentangle the specific impacts of specific types of traumas. Instead, we focused on wider understanding within each theme, with recognition of the relationship between themes rather than looking at direct causal pathways. Finally, despite most papers having reasonable quality levels, over half were identified as only satisfactory due to their overall limited relevancy to the review. Key papers A and B were used for testing the initial a priori coding framework and developing inductive codes, before supplementing the synthesis with the data from the remaining papers.

## Conclusion

The experience of homelessness and its surrounding circumstances are often traumatic. Without appropriate support, the negative impacts on mental health can lead to detrimental management strategies such as substance use. Longer term, this can also lead to people becoming desensitized to trauma and accepting a life where trauma becomes normal. Early intervention can mitigate experiences of trauma and reduce the burden experienced consequently. This is likely to improve the mental health and wellbeing of people who are experiencing homelessness and support their transition out of homelessness. Further research is needed to work with people experiencing homelessness to identify strategies and solutions to address trauma before, during and after it takes place.

## Supplemental Material

sj-docx-1-tva-10.1177_15248380241286839 – Supplemental material for A Co-produced International Qualitative Systematic Review on Lived Experiences of Trauma During Homelessness in Adulthood and Impacts on Mental Health Supplemental material, sj-docx-1-tva-10.1177_15248380241286839 for A Co-produced International Qualitative Systematic Review on Lived Experiences of Trauma During Homelessness in Adulthood and Impacts on Mental Health by Emma A Adams, Kerry Brennan-Tovey, Joanne McGrath, Steven Thirkle, Neha Jain, Maria Raisa Jessica Aquino, Victoria Bartle, Joanne Kennedy, Margaret Ogden, Jeff Parker, Sophie Koehne, Eileen Kaner and Sheena E Ramsay in Trauma, Violence, & Abuse

sj-docx-2-tva-10.1177_15248380241286839 – Supplemental material for A Co-produced International Qualitative Systematic Review on Lived Experiences of Trauma During Homelessness in Adulthood and Impacts on Mental Health Supplemental material, sj-docx-2-tva-10.1177_15248380241286839 for A Co-produced International Qualitative Systematic Review on Lived Experiences of Trauma During Homelessness in Adulthood and Impacts on Mental Health by Emma A Adams, Kerry Brennan-Tovey, Joanne McGrath, Steven Thirkle, Neha Jain, Maria Raisa Jessica Aquino, Victoria Bartle, Joanne Kennedy, Margaret Ogden, Jeff Parker, Sophie Koehne, Eileen Kaner and Sheena E Ramsay in Trauma, Violence, & Abuse
