# Supplementary material for: A Co-produced International Qualitative Systematic Review on Lived Experiences of Trauma During Homelessness in Adulthood and Impacts on Mental Health
Source: Trauma Violence Abuse. 2024 Nov 6;26(3):510–27. doi: 10.1177/15248380241286839 (PMC12145477; doi:10.1177/15248380241286839)
Supplement: sj-docx-1-tva-10.1177_15248380241286839 – Supplemental material for A Co-produced International Qualitative Systematic Review on Lived Experiences of Trauma During Homelessness in Adulthood and Impacts on Mental Health [file sj-docx-1-tva-10.1177_15248380241286839.docx]

**Supplemental Appendix B. Table showing which studies are related to each theme and sub-theme.**

| First Author (Year) | Theme 1: Making sense of homelessness as a trauma | | Theme 2: Dealing with the mental health impacts of trauma | | | | Theme 3: Responses to repeated exposure to trauma | |
| --- | --- | --- | --- | --- | --- | --- | --- | --- |
|  | Experience of homelessness | Chaos surrounding homelessness | A constant state of fear | Anxiety and depression | Substance use as a management strategy | Strategies to feel safe | Acceptance and resignation as a response | Hope for a better tomorrow as a response |
| Biscotto (2016) | P |  | P | P | P | P | P |  |
| Bonugli (2013) |  | P |  | P | P | P | P |  |
| Dickins  (2023) |  |  |  |  | P |  | P |  |
| Elliot  (2018) | P | P |  | P | P | P | P | P |
| Haile  (2020) | P |  |  |  | P |  |  |  |
| Huey  (2012) | P |  |  | P | P |  |  |  |
| Keene  (2013) | P | P | P | P |  | P | P |  |
| Kim  (2004) | P |  | P | P | P |  | P |  |
| Kirkman (2015) | P | P |  |  |  | P |  |  |
| Lewison (2014) | P | P |  |  |  | P |  |  |
| Li  (2020) |  |  | P | P |  |  | P |  |
| McNaughton (2008) |  |  |  |  | P |  |  |  |
| Morrell-Bellai (2000) | P |  | P | P |  |  |  |  |
| Narendorf (2017) | P | P | P | P |  |  |  |  |
| Neale  (2001) |  | P |  | P | P |  | P |  |
| Nettleton (2012) |  |  | P |  | P | P |  |  |
| Nicholls (2021) |  |  |  |  | P |  |  |  |
| Osuji  (2021) | P |  |  |  | P |  | P |  |
| Phipps  (2021) | P |  |  | P |  |  |  | P |
| Piat  (2015) |  | P | P |  |  |  |  |  |
| Rosa  (2015) |  | P | P |  |  | P | P |  |
| Sadeghi  (2021) |  |  | P |  |  | P |  |  |
| Shaikh  (2019) | P |  | P |  | P |  |  |  |
| Silva  (2019) |  |  |  | P |  |  |  |  |
| Sutherland  (2022) | P |  | P | P |  |  |  |  |
| Thomas  (2021) |  | P | P | P | P |  |  |  |
| Williams (2011) |  |  |  |  | P |  |  |  |
