# Supplementary material for: A Co-produced International Qualitative Systematic Review on Lived Experiences of Trauma During Homelessness in Adulthood and Impacts on Mental Health
Source: Trauma Violence Abuse. 2024 Nov 6;26(3):510–27. doi: 10.1177/15248380241286839 (PMC12145477; doi:10.1177/15248380241286839)
Supplement: sj-docx-2-tva-10.1177_15248380241286839 – Supplemental material for A Co-produced International Qualitative Systematic Review on Lived Experiences of Trauma During Homelessness in Adulthood and Impacts on Mental Health [file sj-docx-2-tva-10.1177_15248380241286839.docx]

**Supplemental Appendix A. Full database searches**

**MEDLINE (R) and Epub ahead of print, in process, in-data review, & other non-indexed citations, daily, and versions (1946 to 2024 March 4)**

| 1. | Homeless Persons/ | 10076 |
| --- | --- | --- |
| 2. | (homeless* or undomiciled or houseless or unhoused or vagran* or "street person" or "street people").ti,ab. | 14721 |
| 3. | "rough sleeping".ti,ab. | 30 |
| 4. | ((sleep* adj3 rough) or (temp* accommodation or shelter* or "sofa surf*" or "risk of eviction")).ti,ab. | 15014 |
| 5. | Halfway Houses/ | 1071 |
| 6. | hostel*.ti,ab. | 962 |
| 7. | (night adj3 shelter*).ti,ab. | 64 |
| 8. | (winter adj4 shelter*).ti,ab. | 29 |
| 9. | "emergency accommodation".ti,ab. | 21 |
| 10. | "support accommodation".ti,ab. | 11 |
| 11. | "support* hous*".ti,ab. | 1059 |
| 12. | "no fixed abode".ti,ab. | 56 |
| 13. | "hard to reach".ti,ab. | 2944 |
| 14. | "supported lodging*".ti,ab. | 3 |
| 15. | (housing adj3 (instability or insecurity or loss)).ti,ab. | 1391 |
| 16. | squat*.ti,ab. | 8888 |
| 17. | "bed and breakfast".ti,ab. | 31 |
| 18. | 1 or 2 or 3 or 4 or 5 or 6 or 7 or 8 or 9 or 10 or 11 or 12 or 13 or 14 or 15 or 16 or 17 | 44146 |
| 19. | exp "Wounds and Injuries"/ | 1027491 |
| 20. | trauma*.ti,ab. | 451085 |
| 21. | exp "Psychological Trauma"/ | 2028 |
| 22. | (abuse OR drug*).ti,ab. | 2089925 |
| 23. | exp Violence/ | 115193 |
| 24. | violence.ti,ab. | 65280 |
| 25. | rape.ti,ab. | 9212 |
| 26. | "Sexual Harassment"/ | 2326 |
| 27. | "sexual haras*".ti,ab. | 2406 |
| 28. | "adverse life event*".ti,ab. | 920 |
| 29. | "traumatic event*".ti,ab. | 10401 |
| 30. | neglect.ti,ab. | 25299 |
| 31. | "family breakdown".ti,ab. | 96 |
| 32. | ("substance use" or "substance abuse" or "substance misuse").ti,ab. | 75669 |
| 33. | "Family separation"/ | 82 |
| 34. | Death/ | 20715 |
| 35. | exp Grief/ | 10404 |
| 36. | "serious accident".ti,ab. | 136 |
| 37. | exp "Substance-Related Disorders"/ | 316894 |
| 38. | gang.ti,ab. | 1295 |
| 39. | 19 or 20 or 21 or 22 or 23 or 24 or 25 or 26 or 27 or 28 or 29 or 30 or 31 or 32 or 33 or 34 or 35 or 36 or 37 or 38 | 3644783 |
| 40. | "Mental health"/ | 65877 |
| 41. | ("substance use" or "substance abuse" or "substance misuse").ti,ab. | 75669 |
| 42. | exp "Substance-Related Disorders"/ | 316894 |
| 43. | "mental ill health".ti,ab. | 1427 |
| 44. | "mental health challenges".ti,ab. | 1301 |
| 45. | exp "Anxiety Disorders"/ | 93397 |
| 46. | psychosis.ti,ab. | 44827 |
| 47. | depression/ | 155833 |
| 48. | exp Stress Disorders, Post-Traumatic/ | 42622 |
| 49. | ("post traumatic stress disorder*" or PTSD).ti,ab. | 39722 |
| 50. | exp Schizophrenia/ | 116650 |
| 51. | exp "Personality Disorders"/ | 45761 |
| 52. | Drug*.ti,ab. | 1997936 |
| 53. | 40 or 41 or 42 or 43 or 44 or 45 or 46 or 47 or 48 or 49 or 50 or 51 or 52 | 2697202 |
| 54. | Qualitative Research/ | 85856 |
| 55. | (perceive or perception or perspective or view or experience or attitude or belief or opinion or feel or know or understand or explore or elicit).ti,ab. | 2982891 |
| 56. | (Qualitative or "mixed method*").ti,ab. | 358778 |
| 57. | (Interview or "grounded theory" or ethnography or phenomenolog* or "focus group" or "content analysis" or "thematic analysis" or "constant comparative" or "participant observation").ti,ab. | 324663 |
| 58. | 54 or 55 or 56 or 57 | 3356106 |
| 59. | 18 and 39 and 53 and 58 | 2562 |

**EMBASE 1974 to 2024 March 4**

| 1. | homeless person/ | 3247 |
| --- | --- | --- |
| 2. | (homeless* or undomiciled or houseless or unhoused or vagran* or "street person" or "street people").ti,ab. | 18187 |
| 3. | "rough sleeping".ti,ab. | 40 |
| 4. | ((sleep* adj3 rough) or (temp* accommodation or shelter* or "sofa surf*" or "risk of eviction")).ti,ab. | 17169 |
| 5. | halfway house/ | 1181 |
| 6. | hostel*.ti,ab. | 1398 |
| 7. | (night adj3 shelter*).ti,ab. | 76 |
| 8. | (winter adj4 shelter*).ti,ab. | 31 |
| 9. | "emergency accommodation".ti,ab. | 35 |
| 10. | "support accommodation".ti,ab. | 16 |
| 11. | "no fixed abode".ti,ab. | 82 |
| 12. | "hard to reach".ti,ab. | 3636 |
| 13. | "supported lodging*".ti,ab. | 4 |
| 14. | (housing adj3 (instability or insecurity or loss)).ti,ab. | 1835 |
| 15. | squat*.ti,ab. | 9808 |
| 16. | "bed and breakfast".ti,ab. | 33 |
| 17. | "support* hous*".ti,ab. | 1235 |
| 18. | 1 or 2 or 3 or 4 or 5 or 6 or 7 or 8 or 9 or 10 or 11 or 12 or 13 or 14 or 15 or 16 or 17 | 49918 |
| 19. | exp injury/ | 2684931 |
| 20. | trauma*.ti,ab. | 580697 |
| 21. | exp psychotrauma/ | 11976 |
| 22. | sexual violence/ or violence.ti,ab. or gun violence/ or violence/ or dating violence/ or gender based violence/ or exposure to violence/ or partner violence/ or domestic violence/ or family violence/ or physical violence/ | 108361 |
| 23. | rape.ti,ab. | 9926 |
| 24. | sexual harassment/ | 4078 |
| 25. | "sexual haras*".ti,ab. | 2657 |
| 26. | "adverse life event*".ti,ab. | 1258 |
| 27. | "traumatic event*".ti,ab. | 13230 |
| 28. | self neglect/ or neglect/ | 8669 |
| 29. | "family breakdown".ti,ab. | 131 |
| 30. | drug misuse/ or substance abuse/ or drug dependence/ or exp drug abuse/ | 248333 |
| 31. | family separation/ | 14575 |
| 32. | death/ | 292277 |
| 33. | grief/ or complicated grief/ | 14575 |
| 34. | "serious accident".ti,ab. | 199 |
| 35. | ("substance use" or "substance abuse" or "substance misuse").ti,ab. | 98765 |
| 36. | gang.ti,ab. or gang/ | 1756 |
| 37. | (abuse OR drug*).ti,ab. | 2894365 |
| 38. | 19 or 20 or 21 or 22 or 23 or 24 or 25 or 26 or 27 or 28 or 29 or 30 or 31 or 32 or 33 or 34 or 35 or 36 or 37 | 6023184 |
| 39. | mental health/ | 221764 |
| 40. | drug misuse/ or substance abuse/ or drug dependence/ | 132747 |
| 41. | "substance use"/ | 20493 |
| 42. | ("substance use" or "substance abuse" or "substance misuse").ti,ab. | 98765 |
| 43. | exp drug abuse/ | 142041 |
| 44. | ("mental ill health" or "mental health challenges").ti,ab. | 3067 |
| 45. | anxiety/ or fear/ | 364759 |
| 46. | anxiety disorder/ or mental disease/ or acute stress disorder/ or anxiety neurosis/ or catastrophizing/ or distress syndrome/ or generalized anxiety disorder/ or "mixed anxiety and depression"/ or obsessive compulsive disorder/ or panic/ or phobia/ or posttraumatic stress disorder/ | 546314 |
| 47. | psychosis.ti,ab. | 67761 |
| 48. | depression/ or mood disorder/ | 541626 |
| 49. | ("post traumatic stress disorder*" or "PTSD").ti,ab. | 51532 |
| 50. | schizophrenia/ or psychosis/ | 275568 |
| 51. | personality disorder/ | 32724 |
| 52. | Drug*.ti,ab. | 2773877 |
| 53. | 39 or 40 or 41 or 42 or 43 or 44 or 45 or 46 or 47 or 48 or 49 or 50 or 51 or 52 | 4226017 |
| 54. | video interview/ or interview/ or structured interview/ or semi structured interview/ or audio interview/ or unstructured interview/ or telephone interview/ | 351153 |
| 55. | grounded theory/ or ethnography/ or phenomenology/ or content analysis/ or thematic analysis/ or qualitative research/ or qualitative analysis/ | 265252 |
| 56. | (Interview or "grounded theory" or ethnography or phenomenolog* or "focus group" or "content analysis" or "thematic analysis" or "constant comparative" or "participant observation").ti,ab. | 400925 |
| 57. | (Qualitative or "mixed method*").ti,ab. | 437970 |
| 58. | (perceive or perception or perspective or view or experience or attitude or belief or opinion or feel or know or understand or explore or elicit).ti,ab. | 3923041 |
| 59. | personal experience/ | 67642 |
| 60. | 54 or 55 or 56 or 57 or 58 or 59 | 4530964 |
| 61. | 18 and 38 and 53 and 60 | 3894 |

**APA PsychInfo 1806 to February week 5 2024**

| 1. | homeless/ or homeless mentally ill/ or shelters/ | 10332 |
| --- | --- | --- |
| 2. | (homeless* or undomiciled or houseless or unhoused or vagran* or "street person" or "street people").ti,ab. | 13387 |
| 3. | "rough sleeping".ti,ab. | 32 |
| 4. | ((sleep* adj3 rough) or (temp* accommodation or shelter* or "sofa surf*" or risk of eviction)).ti,ab. | 8996 |
| 5. | Halfway Houses/ | 335 |
| 6. | hostel*.ti,ab. | 669 |
| 7. | (night adj3 shelter*).ti,ab. | 43 |
| 8. | (winter adj4 shelter*).ti,ab. | 9 |
| 9. | "emergency accommodation".ti,ab. | 10 |
| 10. | "support accommodation".ti,ab. | 12 |
| 11. | "support* hous*".ti,ab. | 1041 |
| 12. | "no fixed abode".ti,ab. | 19 |
| 13. | "hard to reach".ti,ab. | 1253 |
| 14. | "supported lodging*".ti,ab. | 2 |
| 15. | (housing adj3 (instability or insecurity or loss)).ti,ab. | 837 |
| 16. | squat*.ti,ab. | 656 |
| 17. | "bed and breakfast".ti,ab. | 26 |
| 18. | 1 or 2 or 3 or 4 or 5 or 6 or 7 or 8 or 9 or 10 or 11 or 12 or 13 or 14 or 15 or 16 or 17 | 24528 |
| 19. | Injuries/ or Head Injuries/ | 20435 |
| 20. | exp Wounds/ | 1455 |
| 21. | exp Emotional Trauma/ or exp Trauma/ | 83586 |
| 22. | trauma*.ti,ab. | 135748 |
| 23. | exp Physical Abuse/ or exp Verbal Abuse/ or abuse.ti,ab. or exp Sexual Abuse/ | 135912 |
| 24. | exp Violence/ | 128448 |
| 25. | rape/ or sexual abuse/ | 29097 |
| 26. | violent crime/ | 2839 |
| 27. | "sexual haras*".ti,ab. | 3896 |
| 28. | "adverse life event*".ti,ab. | 899 |
| 29. | "traumatic event*".ti,ab. | 12662 |
| 30. | neglect.ti,ab. | 25447 |
| 31. | "family breakdown".ti,ab. | 199 |
| 32. | drug abuse/ or inhalant abuse/ or polydrug abuse/ or drug overdoses/ | 54377 |
| 33. | ("substance misuse" or "substance use" or "substance abuse").ti,ab. | 80448 |
| 34. | family separation/ | 293 |
| 35. | "death and dying"/ or parental death/ or partner death/ or traumatic loss/ | 39859 |
| 36. | exp Grief/ | 14945 |
| 37. | "serious accident".ti,ab. | 78 |
| 38. | Drug*.ti,ab. | 231856 |
| 39. | exp Gangs/ or gang.ti,ab. | 3656 |
| 40. | 19 or 20 or 21 or 22 or 23 or 24 or 25 or 26 or 27 or 28 or 29 or 30 or 31 or 32 or 33 or 34 or 35 or 36 or 37 or 38 or 39 | 639006 |
| 41. | drug abuse/ or inhalant abuse/ or polydrug abuse/ or drug overdoses/ | 54377 |
| 42. | Mental Health/ | 95120 |
| 43. | exp "Substance Use Disorder"/ | 125631 |
| 44. | ("substance use" or "substance abuse" or "substance misuse").ti,ab. | 80448 |
| 45. | "mental ill health".ti,ab. | 1291 |
| 46. | "mental health challenges".ti,ab. | 1311 |
| 47. | exp anxiety/ or anxiety disorders/ or fear/ or generalized anxiety disorder/ or panic/ or panic attack/ or panic disorder/ or posttraumatic stress/ or trauma reactions/ | 141018 |
| 48. | exp Psychosis/ | 131021 |
| 49. | affective disorders/ or mental disorders/ or disruptive mood dysregulation disorder/ or major depression/ or seasonal affective disorder/ | 262291 |
| 50. | ("post traumatic stress disorder*" or PTSD).ti,ab. | 4478 |
| 51. | exp Schizophrenia/ | 100852 |
| 52. | exp Personality Disorders/ | 32754 |
| 53. | Drug*.ti,ab. | 231856 |
| 54. | 41 or 42 or 43 or 44 or 45 or 46 or 47 or 48 or 49 or 50 or 51 or 52 or 53 | 892974 |
| 55. | qualitative methods/ or focus group/ or grounded theory/ or interpretative phenomenological analysis/ or narrative analysis/ or semi-structured interview/ or thematic analysis/ or mixed methods research/ or phenomenology/ | 40141 |
| 56. | ethnography/ or content analysis/ or discourse analysis/ or narrative analysis/ | 28688 |
| 57. | (Interview or "grounded theory" or ethnography or phenomenolog* or "focus group" or "content analysis" or "thematic analysis" or "constant comparative" or "participant observation").ti,ab. | 276994 |
| 58. | (Qualitative or "mixed method*").ti,ab. | 241720 |
| 59. | (perceive or perception or perspective or view or experience or attitude or belief or opinion or feel or know or understand or explore or elicit).ti,ab. | 1444197 |
| 60. | 55 or 56 or 57 or 58 or 59 | 1681041 |
| 61. | 18 and 40 and 54 and 60 | 2356 |

**SCOPUS (ran on 5 March 2024) – 1961 was launch of database**

TITLE-ABS ( homeless*  OR  undomiciled  OR  houseless  OR  unhoused  OR  vagran*  OR  "street person"  OR  "street people"  OR  "temp* accommodation"  OR  shelter*  OR  "sofa surf*"  OR  "risk of eviction"  OR  "rough sleeping"  OR  "sleep* W/4 rough"  OR  "halfway house*"  OR  hostel*  OR  "night W/4 shelter*"  OR  "winter W/5 shelter*"  OR  "emergency accommodation"  OR  "support accommodation"  OR  "no fixed abode"  OR  "hard to reach"  OR  "supported lodging*"  OR  "housing W/4 instability"  OR  "housing W/4 insecurity"  OR  "housing W/4 loss"  OR  squat*  OR  "bed and breakfast"  OR  "support* hous*" )  AND  TITLE-ABS ( wound*  OR  injur*  OR  trauma*  OR  "psycho* trauma"  OR  abuse  OR  violence  OR  rape  OR  "sexual haras*"  OR  "adverse life events"  OR  "traumatic event"  OR  neglect  OR  "famil* breakdown"  OR  "substance use" OR “substance misuse” OR “substance abuse”  OR  drug  OR  "family separation"  OR  death  OR  grief  OR  "serious accident"  OR  gang  OR  abuse )  AND  TITLE-ABS ( "mental health"  OR  "substance use" OR “substance misuse” OR “substance abuse”  OR  drug  OR  "mental ill health"  OR  anxiety  OR  anxious  OR  fear  OR  stress  OR  depression  OR  panic  OR  phobia  OR  "posttraumatic stress"  OR PTSD OR  psychosis  OR  "psychotic disorder"  OR  "mood disorder"  OR  "post traumatic stress"  OR  schizophrenia  OR  "personality disorder"  OR  alcohol )  AND  TITLE-ABS ( interview  OR  "grounded theory"  OR  ethnography  OR  phenomenology  OR  "content analysis"  OR  "thematic analysis"  OR  qualitative  OR  "focus group"  OR  perception  OR  attitude  OR  experience  OR  "mixed method*" )

*3580 documents found*

**Web of Science (ran on 5 March 2024)**

(TI=(homeless* OR undomiciled OR houseless OR unhoused OR vagran* OR "street person" OR "street people" OR "temp* accommodation" OR shelter* OR "sofa surf*" OR "risk of eviction" OR "rough sleeping" OR "sleep* NEAR/3 rough" OR "halfway house*" OR hostel* OR "night NEAR/3 shelter*" OR "winter NEAR/4 shelter*" OR "emergency accommodation" OR "support accommodation" OR "no fixed abode" OR "hard to reach" OR "supported lodging*" OR "housing NEAR/3 instability" OR "housing NEAR/3 insecurity" OR "housing NEAR/3 loss" OR squat* OR "bed and breakfast" OR "support* hous*" ) OR AB=(homeless* OR undomiciled OR houseless OR unhoused OR vagran* OR "street person" OR "street people" OR "temp* accommodation" OR shelter* OR "sofa surf*" OR "risk of eviction" OR "rough sleeping" OR "sleep* NEAR/3 rough" OR "halfway house*" OR hostel* OR "night NEAR/3 shelter*" OR "winter NEAR/4 shelter*" OR "emergency accommodation" OR "support accommodation" OR "no fixed abode" OR "hard to reach" OR "supported lodging*" OR "housing NEAR/3 instability" OR "housing NEAR/3 insecurity" OR "housing NEAR/3 loss" OR squat* OR "bed and breakfast" OR "support* hous*"))

AND  (TI=( wound*  OR  injur*  OR  trauma*  OR  "psycho* trauma"  OR  abuse  OR  violence  OR  rape  OR  "sexual haras*"  OR  "adverse life events"  OR  "traumatic event"  OR  neglect  OR  "famil* breakdown"  OR  "substance use" OR “substance misuse” OR “substance abuse”  OR  drug  OR  "family separation"  OR  death  OR  grief  OR  "serious accident"  OR  gang  OR  abuse )  OR AB=( wound*  OR  injur*  OR  trauma*  OR  "psycho* trauma"  OR  abuse  OR  violence  OR  rape  OR  "sexual haras*"  OR  "adverse life events"  OR  "traumatic event"  OR  neglect  OR  "famil* breakdown"  OR  "substance use"  OR “substance misuse” OR “substance abuse”  OR  drug  OR  "family separation"  OR  death  OR  grief  OR  "serious accident" OR  gang  OR  abuse ) )

AND (TI=( "mental health"  OR  "substance use" OR “substance misuse” OR “substance abuse”  OR  drug  OR  "mental ill health"  OR  anxiety  OR  anxious  OR  fear  OR  stress  OR  depression  OR  panic  OR  phobia  OR  "posttraumatic stress"  OR PTSD OR  psychosis  OR  "psychotic disorder"  OR  "mood disorder"  OR  "post traumatic stress"  OR  schizophrenia  OR  "personality disorder"  OR  alcohol ) OR AB=( "mental health"  OR  "substance use" OR “substance misuse” OR “substance abuse”    OR  drug  OR  "mental ill health"  OR  anxiety  OR  anxious  OR  fear  OR  stress  OR  depression  OR  panic  OR  phobia  OR  "posttraumatic stress"  OR PTSD OR  psychosis  OR  "psychotic disorder"  OR  "mood disorder"  OR  "post traumatic stress"  OR  schizophrenia  OR  "personality disorder"  OR  alcohol ) )

AND (TI=( interview  OR  "grounded theory"  OR  ethnography  OR  phenomenology  OR  "content analysis"  OR  "thematic analysis"  OR  qualitative  OR  "focus group"  OR  perception  OR  attitude  OR  experience  OR  "mixed methods" ) OR AB=( interview  OR  "grounded theory"  OR  ethnography  OR  phenomenology  OR  "content analysis"  OR  "thematic analysis"  OR  qualitative  OR  "focus group"  OR  perception  OR  attitude  OR  experience  OR  "mixed method*" ))

*3857 results from web of science core collection*

**CINAHL (ran 5 March 2024)**

|  | (MH "Homeless Persons") OR (MH "Homelessness") OR (MH "Halfway Houses") OR (MH "Housing Instability") | Title  11,118 |
| --- | --- | --- |
|  | (MH "Homeless Persons") OR (MH "Homelessness") OR (MH "Halfway Houses") OR (MH "Housing Instability") | Abstract  11,118 |
|  | Homeless* OR undomiciled OR houseless OR unhoused OR vagran* OR "street person" OR "street people" OR "rough sleeping” OR “sleep* N3 rough” OR “temp* accommodation” OR shelter* OR “sofa surf*” OR “risk of eviction” OR hostel* OR “night W3 shelter” OR “winter W4 shelter” OR “emergency accommodation” OR “support accommodation” OR “no fixed abode” OR “hard to reach” OR “supported lodging” OR “housing W3 instability” OR “housing W3 insecurity” OR “housing W3 loss” OR Squat* OR “bed and breakfast” OR “support* hous*” | Title  6,899 |
|  | Homeless* OR undomiciled OR houseless OR unhoused OR vagran* OR "street person" OR "street people" OR "rough sleeping” OR “sleep* N3 rough” OR “temp* accommodation” OR shelter* OR “sofa surf*” OR “risk of eviction” OR hostel* OR “night W3 shelter” OR “winter W4 shelter” OR “emergency accommodation” OR “support accommodation” OR “no fixed abode” OR “hard to reach” OR “supported lodging” OR “housing W3 instability” OR “housing W3 insecurity” OR “housing W3 loss” OR Squat* OR “bed and breakfast” OR “support* hous*” | Abstract  11,920 |
|  | Combine pop lines  S1 OR S2 OR S3 OR S4 | 19,621 |
|  | (MH "Trauma") OR (MH "Microtrauma") OR (MH "Multiple Trauma") OR (MH "Wounds and Injuries+") OR (MH "Historical Trauma") OR (MH "Sexual Trauma") OR (MH "Stress, Psychological") OR (MH "Psychological Trauma") OR (MH "Violence") OR (MH "Adverse Childhood Experiences") OR (MH "Dating Violence") OR (MH "Domestic Violence") OR (MH "Exposure to Violence") OR (MH "Gender-Based Violence") OR (MH "Gun Violence") OR (MH "Sexual Abuse") OR (MH "Rape") or (MH “Sexual Harassment”) OR (MH "Neglect (Omaha)") OR (MH "Self Neglect") OR (MH "Substance Abusers") OR (MH "Substance Abuse") OR (MH "Alcohol-Related Disorders") OR (MH "Inhalant Abuse") OR (MH "Substance Abuse, Intravenous") OR (MH "Substance Abuse, Perinatal") OR (MH “Dysfunctional Family”) OR (MH “Death”) OR (MH "Grief (Omaha)") OR (MH "Complicated Grief") OR (MH "Grief") OR (MH “Gangs”) | Title  530,066 |
|  | (MH "Trauma") OR (MH "Microtrauma") OR (MH "Multiple Trauma") OR (MH "Wounds and Injuries+") OR (MH "Historical Trauma") OR (MH "Sexual Trauma") OR (MH "Stress, Psychological") OR (MH "Psychological Trauma") OR (MH "Violence") OR (MH "Adverse Childhood Experiences") OR (MH "Dating Violence") OR (MH "Domestic Violence") OR (MH "Exposure to Violence") OR (MH "Gender-Based Violence") OR (MH "Gun Violence") OR (MH "Sexual Abuse") OR (MH "Rape") or (MH “Sexual Harassment”) OR (MH "Neglect (Omaha)") OR (MH "Self Neglect") OR (MH "Substance Abusers") OR (MH "Substance Abuse") OR (MH "Alcohol-Related Disorders") OR (MH "Inhalant Abuse") OR (MH "Substance Abuse, Intravenous") OR (MH "Substance Abuse, Perinatal") OR (MH “Dysfunctional Family”) OR (MH “Death”) OR (MH "Grief (Omaha)") OR (MH "Complicated Grief") OR (MH "Grief") OR (MH “Gangs”) | abstract  530,066 |
|  | Trauma* OR rape OR “sexual haras*” OR “traumatic event*” OR “adverse life events” OR “family breakdown” OR abuse OR drug OR “substance use” OR “substance abuse” OR “substance misuse” | Title  67,694 |
|  | Trauma* OR rape OR “sexual haras*” OR “traumatic event*” OR “adverse life events” OR “family breakdown” OR abuse OR drug OR “substance use” OR “substance abuse” OR “substance misuse” | abstract  123,608 |
|  | Combine phenomenon of interest lines  S6 OR S7 OR S8 OR S9 | 613,133 |
|  | (MH "Mental Health") OR (MH "Mental Health (Omaha)") OR (MH "Mental Disorders+") OR (MH "Substance Abusers") OR (MH "Substance Abuse") OR (MH "Alcohol-Related Disorders") OR (MH "Inhalant Abuse") OR (MH "Substance Abuse, Intravenous") OR (MH "Substance Abuse, Perinatal") OR (MH "Alcoholics") OR (MH "Intravenous Drug Users") OR (MH "Anxiety") OR (MH "Anxiety (Saba CCC)") | Title  727,057 |
|  | (MH "Mental Health") OR (MH "Mental Health (Omaha)") OR (MH "Mental Disorders+") OR (MH "Substance Abusers") OR (MH "Substance Abuse") OR (MH "Alcohol-Related Disorders") OR (MH "Inhalant Abuse") OR (MH "Substance Abuse, Intravenous") OR (MH "Substance Abuse, Perinatal") OR (MH "Alcoholics") OR (MH "Intravenous Drug Users") OR (MH "Anxiety") OR (MH "Anxiety (Saba CCC)") | Abstract  727,057 |
|  | “substance use” OR “substance abuse” OR “substance misuse” OR “mental health” OR “mental ill health” OR “post traumatic stress” OR PTSD OR drug | Title  182,436 |
|  | “substance use” OR “substance abuse” OR “substance misuse” OR “mental health” OR “mental ill health” OR “post traumatic stress” OR PTSD OR drug | Abstract  371,122 |
|  | Combine outcomes  S11 OR S12 OR S13 OR S14 | 1,041,612 |
|  | (MH "Qualitative Studies+") OR (MH "Multimethod Studies") OR (MH "Grounded Theory") OR (MH "Action Research") OR (MH "Ethnographic Research") OR (MH "Exploratory Research") OR (MH “Phenomenological Research”) OR (MH "Focus Groups") OR (MH "Interviews+") OR (MH "Narratives") OR (MH "Observational Methods") OR (MH "Audiorecording") OR (MH "Constant Comparative Method") OR (MH "Content Analysis") OR (MH "Discourse Analysis") OR (MH "Thematic Analysis") | Title  435,192 |
|  | MH "Qualitative Studies+") OR (MH "Multimethod Studies") OR (MH "Grounded Theory") OR (MH "Action Research") OR (MH "Ethnographic Research") OR (MH "Exploratory Research") OR (MH “Phenomenological Research”) OR (MH "Focus Groups") OR (MH "Interviews+") OR (MH "Narratives") OR (MH "Observational Methods") OR (MH "Audiorecording") OR (MH "Constant Comparative Method") OR (MH "Content Analysis") OR (MH "Discourse Analysis") OR (MH "Thematic Analysis") | Abstract  435,192 |
|  | Interview OR "grounded theory" OR ethnography OR phenomenolo* OR "focus group" OR "content analysis" OR "thematic analysis" OR "constant comparative" OR "participant observation" OR “qualitative” OR “mixed metho*” OR perceive OR perception OR perspective OR view OR experience OR attitude OR belief OR opinion OR feel OR know OR understand OR explore OR elicit | Title  415,282 |
|  | Interview OR "grounded theory" OR ethnography OR phenomenolo* OR "focus group" OR "content analysis" OR "thematic analysis" OR "constant comparative" OR "participant observation" OR “qualitative” OR “mixed metho*” OR perceive OR perception OR perspective OR view OR experience OR attitude OR belief OR opinion OR feel OR know OR understand OR explore OR elicit | Abstract  1,028,887 |
|  | Combine design  S16 OR S17 OR S18 OR S19 | 1,372,919 |
|  | Combine all  S5 AND S10 AND S15 AND S20 | 1,530 |

**ASSIA (Proquest) (ran of 5^th^ March 2024)**

(((MAINSUBJECT.EXACT("Homeless men") OR MAINSUBJECT.EXACT("Homeless mentally ill men") OR MAINSUBJECT.EXACT("Homeless people") OR MAINSUBJECT.EXACT("Homeless mentally ill young people") OR MAINSUBJECT.EXACT("Homeless young people") OR MAINSUBJECT.EXACT("Homeless mentally ill people") OR MAINSUBJECT.EXACT("Homelessness") OR MAINSUBJECT.EXACT("Homeless elderly people") OR MAINSUBJECT.EXACT("Homeless mentally ill women") OR MAINSUBJECT.EXACT("Homeless young men") OR MAINSUBJECT.EXACT("Homeless mothers") OR MAINSUBJECT.EXACT("Homeless families") OR MAINSUBJECT.EXACT("Homeless women") OR MAINSUBJECT.EXACT("Homeless young women") OR MAINSUBJECT.EXACT("Homeless pregnant women") OR MAINSUBJECT.EXACT("Homeless older people") OR MAINSUBJECT.EXACT("Intentional homelessness") OR MAINSUBJECT.EXACT("Halfway houses")) OR (MAINSUBJECT.EXACT("Homeless men") OR MAINSUBJECT.EXACT("Homeless mentally ill men") OR MAINSUBJECT.EXACT("Homeless people") OR MAINSUBJECT.EXACT("Homeless mentally ill young people") OR MAINSUBJECT.EXACT("Homeless young people") OR MAINSUBJECT.EXACT("Homeless mentally ill people") OR MAINSUBJECT.EXACT("Homelessness") OR MAINSUBJECT.EXACT("Homeless elderly people") OR MAINSUBJECT.EXACT("Homeless mentally ill women") OR MAINSUBJECT.EXACT("Homeless young men") OR MAINSUBJECT.EXACT("Homeless mothers") OR MAINSUBJECT.EXACT("Homeless families") OR MAINSUBJECT.EXACT("Homeless women") OR MAINSUBJECT.EXACT("Homeless young women") OR MAINSUBJECT.EXACT("Homeless pregnant women") OR MAINSUBJECT.EXACT("Homeless older people") OR MAINSUBJECT.EXACT("Intentional homelessness") OR MAINSUBJECT.EXACT("Halfway houses"))) OR (ab(homeless* OR undomiciled OR houseless OR unhoused OR vagran* OR "street person" OR "street people" OR "rough sleeping" OR "temp* accommodation" OR shelter* OR "sofa surf*" OR "risk of eviction" OR hostel* OR "emergency accommodation" OR "support accommodation" OR "no fixed abode" OR "hard to reach" OR "supported lodging" OR Squat* OR "bed and breakfast" OR "support* hous*") OR ti(homeless* OR undomiciled OR houseless OR unhoused OR vagran* OR "street person" OR "street people" OR "rough sleeping" OR "temp* accommodation" OR shelter* OR "sofa surf*" OR "risk of eviction" OR hostel* OR "emergency accommodation" OR "support accommodation" OR "no fixed abode" OR "hard to reach" OR "supported lodging" OR Squat* OR "bed and breakfast" OR "support* hous*")) OR (ab("housing NEAR/3 instability" OR "housing NEAR/3 insecurity" OR "housing NEAR/3 loss" OR "sleep* NEAR/3 rough" OR "night NEAR/3 shelter" OR "winter NEAR/4 shelter") OR ti("housing NEAR/3 instability" OR "housing NEAR/3 insecurity" OR "housing NEAR/3 loss" OR "sleep* NEAR/3 rough" OR "night NEAR/3 shelter" OR "winter NEAR/4 shelter"))) AND (((MAINSUBJECT.EXACT("Injuries") OR MAINSUBJECT.EXACT("Chronic posttraumatic stress disorder") OR MAINSUBJECT.EXACT("Psychological trauma") OR MAINSUBJECT.EXACT("Repetitive trauma") OR MAINSUBJECT.EXACT("Traumatic life events") OR MAINSUBJECT.EXACT("Vicarious trauma") OR MAINSUBJECT.EXACT("Alcohol related trauma") OR MAINSUBJECT.EXACT("Traumatic grief") OR MAINSUBJECT.EXACT("Posttraumatic stress disorder") OR MAINSUBJECT.EXACT("Traumatic stress") OR MAINSUBJECT.EXACT("Traumatic incidents") OR MAINSUBJECT.EXACT("Combat related posttraumatic stress disorder") OR MAINSUBJECT.EXACT("Physical trauma") OR MAINSUBJECT.EXACT("Violence") OR MAINSUBJECT.EXACT("Domestic violence") OR MAINSUBJECT.EXACT("Sexual violence") OR MAINSUBJECT.EXACT("Rape") OR MAINSUBJECT.EXACT("Family breakdown") OR MAINSUBJECT.EXACT("Gangs") OR MAINSUBJECT.EXACT("Harassment") OR MAINSUBJECT.EXACT("Sexual harassment") OR MAINSUBJECT.EXACT("Sexual abuse") OR MAINSUBJECT.EXACT("Traumatic grief") OR MAINSUBJECT.EXACT("Neglect") OR MAINSUBJECT.EXACT("Death") OR MAINSUBJECT.EXACT("Grief") OR MAINSUBJECT.EXACT("Bereavement") OR MAINSUBJECT.EXACT("Drug abusers") OR MAINSUBJECT.EXACT("Drug addiction") OR MAINSUBJECT.EXACT("Drug addicts") OR MAINSUBJECT.EXACT("Drug dependency units") OR MAINSUBJECT.EXACT("Overdoses") OR MAINSUBJECT.EXACT("Alcohol abuse") OR MAINSUBJECT.EXACT("Substance abuse disorders") OR MAINSUBJECT.EXACT("Drug abuse") OR MAINSUBJECT.EXACT("Substance abuse")) OR (MAINSUBJECT.EXACT("Injuries") OR MAINSUBJECT.EXACT("Chronic posttraumatic stress disorder") OR MAINSUBJECT.EXACT("Psychological trauma") OR MAINSUBJECT.EXACT("Repetitive trauma") OR MAINSUBJECT.EXACT("Traumatic life events") OR MAINSUBJECT.EXACT("Vicarious trauma") OR MAINSUBJECT.EXACT("Alcohol related trauma") OR MAINSUBJECT.EXACT("Traumatic grief") OR MAINSUBJECT.EXACT("Posttraumatic stress disorder") OR MAINSUBJECT.EXACT("Traumatic stress") OR MAINSUBJECT.EXACT("Traumatic incidents") OR MAINSUBJECT.EXACT("Combat related posttraumatic stress disorder") OR MAINSUBJECT.EXACT("Physical trauma") OR MAINSUBJECT.EXACT("Violence") OR MAINSUBJECT.EXACT("Domestic violence") OR MAINSUBJECT.EXACT("Sexual violence") OR MAINSUBJECT.EXACT("Rape") OR MAINSUBJECT.EXACT("Family breakdown") OR MAINSUBJECT.EXACT("Gangs") OR MAINSUBJECT.EXACT("Harassment") OR MAINSUBJECT.EXACT("Sexual harassment") OR MAINSUBJECT.EXACT("Sexual abuse") OR MAINSUBJECT.EXACT("Traumatic grief") OR MAINSUBJECT.EXACT("Neglect") OR MAINSUBJECT.EXACT("Death") OR MAINSUBJECT.EXACT("Grief") OR MAINSUBJECT.EXACT("Bereavement") OR MAINSUBJECT.EXACT("Drug abusers") OR MAINSUBJECT.EXACT("Drug addiction") OR MAINSUBJECT.EXACT("Drug addicts") OR MAINSUBJECT.EXACT("Drug dependency units") OR MAINSUBJECT.EXACT("Overdoses") OR MAINSUBJECT.EXACT("Alcohol abuse") OR MAINSUBJECT.EXACT("Substance abuse disorders") OR MAINSUBJECT.EXACT("Drug abuse") OR MAINSUBJECT.EXACT("Substance abuse"))) OR (ab(trauma* OR rape OR "sexual haras*" OR "traumatic event" OR "adverse life events" OR "family breakdown" OR abuse OR "substance use" OR "substance misuse" OR "substance abuse" OR drug*) OR ti(trauma* OR rape OR "sexual haras*" OR "traumatic event" OR "adverse life events" OR "family breakdown" OR abuse OR "substance use" OR "substance misuse" OR "substance abuse" OR drug*))) AND (((MAINSUBJECT.EXACT("Personality disorders") OR MAINSUBJECT.EXACT("Behaviour disorders") OR MAINSUBJECT.EXACT("Mental health") OR MAINSUBJECT.EXACT("Psychiatric disorders") OR MAINSUBJECT.EXACT("Anxiety disorders") OR MAINSUBJECT.EXACT("Psychotic mood disorders") OR MAINSUBJECT.EXACT("Schizophrenia") OR MAINSUBJECT.EXACT("Mental illness") OR MAINSUBJECT.EXACT("Drug abusers") OR MAINSUBJECT.EXACT("Drug addiction") OR MAINSUBJECT.EXACT("Drug addicts") OR MAINSUBJECT.EXACT("Drug dependency units") OR MAINSUBJECT.EXACT("Overdoses") OR MAINSUBJECT.EXACT("Alcohol abuse") OR MAINSUBJECT.EXACT("Substance abuse disorders") OR MAINSUBJECT.EXACT("Drug abuse") OR MAINSUBJECT.EXACT("Substance abuse")) OR (MAINSUBJECT.EXACT("Personality disorders") OR MAINSUBJECT.EXACT("Behaviour disorders") OR MAINSUBJECT.EXACT("Mental health") OR MAINSUBJECT.EXACT("Psychiatric disorders") OR MAINSUBJECT.EXACT("Anxiety disorders") OR MAINSUBJECT.EXACT("Psychotic mood disorders") OR MAINSUBJECT.EXACT("Schizophrenia") OR MAINSUBJECT.EXACT("Mental illness") OR MAINSUBJECT.EXACT("Drug abusers") OR MAINSUBJECT.EXACT("Drug addiction") OR MAINSUBJECT.EXACT("Drug addicts") OR MAINSUBJECT.EXACT("Drug dependency units") OR MAINSUBJECT.EXACT("Overdoses") OR MAINSUBJECT.EXACT("Alcohol abuse") OR MAINSUBJECT.EXACT("Substance abuse disorders") OR MAINSUBJECT.EXACT("Drug abuse") OR MAINSUBJECT.EXACT("Substance abuse"))) OR (ab("substance use" OR "substance abuse" OR "substance misuse" OR "mental health" OR "mental ill health" OR "post traumatic stress" OR drug OR PTSD) OR ti("substance use" OR "substance abuse" OR "substance misuse" OR "mental health" OR "mental ill health" OR "post traumatic stress" OR drug OR PTSD))) AND (((MAINSUBJECT.EXACT("Qualitative data") OR MAINSUBJECT.EXACT("Qualitative methods") OR MAINSUBJECT.EXACT("Qualitative analysis") OR MAINSUBJECT.EXACT("Qualitative research") OR MAINSUBJECT.EXACT("Content analysis") OR MAINSUBJECT.EXACT("Observational research") OR MAINSUBJECT.EXACT("Interpretative phenomenological analysis") OR MAINSUBJECT.EXACT("Discourse analysis") OR MAINSUBJECT.EXACT("Multimethod research") OR MAINSUBJECT.EXACT("Focus group interviews") OR MAINSUBJECT.EXACT("Interviews") OR MAINSUBJECT.EXACT("Grounded theory") OR MAINSUBJECT.EXACT("Phenomenology") OR MAINSUBJECT.EXACT("Action research") OR MAINSUBJECT.EXACT("Ethnography") OR MAINSUBJECT.EXACT("Narratives") OR MAINSUBJECT.EXACT("Semistructured interviews")) OR (MAINSUBJECT.EXACT("Qualitative data") OR MAINSUBJECT.EXACT("Qualitative methods") OR MAINSUBJECT.EXACT("Qualitative analysis") OR MAINSUBJECT.EXACT("Qualitative research") OR MAINSUBJECT.EXACT("Content analysis") OR MAINSUBJECT.EXACT("Observational research") OR MAINSUBJECT.EXACT("Interpretative phenomenological analysis") OR MAINSUBJECT.EXACT("Discourse analysis") OR MAINSUBJECT.EXACT("Multimethod research") OR MAINSUBJECT.EXACT("Focus group interviews") OR MAINSUBJECT.EXACT("Interviews") OR MAINSUBJECT.EXACT("Grounded theory") OR MAINSUBJECT.EXACT("Phenomenology") OR MAINSUBJECT.EXACT("Action research") OR MAINSUBJECT.EXACT("Ethnography") OR MAINSUBJECT.EXACT("Narratives") OR MAINSUBJECT.EXACT("Semistructured interviews"))) OR (ab(Interview OR "grounded theory" OR ethnography OR phenomenolo* OR "focus group" OR "content analysis" OR "thematic analysis" OR "constant comparative" OR "participant observation" OR "qualitative" OR "mixed method*" OR perceive OR perception OR perspective OR view OR experience OR attitude OR belief OR opinion OR feel OR know OR understand OR explore OR elicit) OR ti(Interview OR "grounded theory" OR ethnography OR phenomenolo* OR "focus group" OR "content analysis" OR "thematic analysis" OR "constant comparative" OR "participant observation" OR "qualitative" OR "mixed method*" OR perceive OR perception OR perspective OR view OR experience OR attitude OR belief OR opinion OR feel OR know OR understand OR explore OR elicit)))

*1,253 results*

**Proquest Dissertations & Theses Global (ran 5 March 2024)**

[**https://about.proquest.com/en/products-services/pqdtglobal/**](https://about.proquest.com/en/products-services/pqdtglobal/)

Searched using command line, following command inserted:

(((MAINSUBJECT.EXACT("Homeless men") OR MAINSUBJECT.EXACT("Homeless mentally ill men") OR MAINSUBJECT.EXACT("Homeless people") OR MAINSUBJECT.EXACT("Homeless mentally ill young people") OR MAINSUBJECT.EXACT("Homeless young people") OR MAINSUBJECT.EXACT("Homeless mentally ill people") OR MAINSUBJECT.EXACT("Homelessness") OR MAINSUBJECT.EXACT("Homeless elderly people") OR MAINSUBJECT.EXACT("Homeless mentally ill women") OR MAINSUBJECT.EXACT("Homeless young men") OR MAINSUBJECT.EXACT("Homeless mothers") OR MAINSUBJECT.EXACT("Homeless families") OR MAINSUBJECT.EXACT("Homeless women") OR MAINSUBJECT.EXACT("Homeless young women") OR MAINSUBJECT.EXACT("Homeless pregnant women") OR MAINSUBJECT.EXACT("Homeless older people") OR MAINSUBJECT.EXACT("Intentional homelessness") OR MAINSUBJECT.EXACT("Halfway houses")) OR (MAINSUBJECT.EXACT("Homeless men") OR MAINSUBJECT.EXACT("Homeless mentally ill men") OR MAINSUBJECT.EXACT("Homeless people") OR MAINSUBJECT.EXACT("Homeless mentally ill young people") OR MAINSUBJECT.EXACT("Homeless young people") OR MAINSUBJECT.EXACT("Homeless mentally ill people") OR MAINSUBJECT.EXACT("Homelessness") OR MAINSUBJECT.EXACT("Homeless elderly people") OR MAINSUBJECT.EXACT("Homeless mentally ill women") OR MAINSUBJECT.EXACT("Homeless young men") OR MAINSUBJECT.EXACT("Homeless mothers") OR MAINSUBJECT.EXACT("Homeless families") OR MAINSUBJECT.EXACT("Homeless women") OR MAINSUBJECT.EXACT("Homeless young women") OR MAINSUBJECT.EXACT("Homeless pregnant women") OR MAINSUBJECT.EXACT("Homeless older people") OR MAINSUBJECT.EXACT("Intentional homelessness") OR MAINSUBJECT.EXACT("Halfway houses"))) OR (ab(homeless* OR undomiciled OR houseless OR unhoused OR vagran* OR "street person" OR "street people" OR "rough sleeping" OR "temp* accommodation" OR shelter* OR "sofa surf*" OR "risk of eviction" OR hostel* OR "emergency accommodation" OR "support accommodation" OR "no fixed abode" OR "hard to reach" OR "supported lodging" OR Squat* OR "bed and breakfast" OR "support* hous*") OR ti(homeless* OR undomiciled OR houseless OR unhoused OR vagran* OR "street person" OR "street people" OR "rough sleeping" OR "temp* accommodation" OR shelter* OR "sofa surf*" OR "risk of eviction" OR hostel* OR "emergency accommodation" OR "support accommodation" OR "no fixed abode" OR "hard to reach" OR "supported lodging" OR Squat* OR "bed and breakfast" OR "support* hous*")) OR (ab("housing NEAR/3 instability" OR "housing NEAR/3 insecurity" OR "housing NEAR/3 loss" OR "sleep* NEAR/3 rough" OR "night NEAR/3 shelter" OR "winter NEAR/4 shelter") OR ti("housing NEAR/3 instability" OR "housing NEAR/3 insecurity" OR "housing NEAR/3 loss" OR "sleep* NEAR/3 rough" OR "night NEAR/3 shelter" OR "winter NEAR/4 shelter"))) AND (((MAINSUBJECT.EXACT("Injuries") OR MAINSUBJECT.EXACT("Chronic posttraumatic stress disorder") OR MAINSUBJECT.EXACT("Psychological trauma") OR MAINSUBJECT.EXACT("Repetitive trauma") OR MAINSUBJECT.EXACT("Traumatic life events") OR MAINSUBJECT.EXACT("Vicarious trauma") OR MAINSUBJECT.EXACT("Alcohol related trauma") OR MAINSUBJECT.EXACT("Traumatic grief") OR MAINSUBJECT.EXACT("Posttraumatic stress disorder") OR MAINSUBJECT.EXACT("Traumatic stress") OR MAINSUBJECT.EXACT("Traumatic incidents") OR MAINSUBJECT.EXACT("Combat related posttraumatic stress disorder") OR MAINSUBJECT.EXACT("Physical trauma") OR MAINSUBJECT.EXACT("Violence") OR MAINSUBJECT.EXACT("Domestic violence") OR MAINSUBJECT.EXACT("Sexual violence") OR MAINSUBJECT.EXACT("Rape") OR MAINSUBJECT.EXACT("Family breakdown") OR MAINSUBJECT.EXACT("Gangs") OR MAINSUBJECT.EXACT("Harassment") OR MAINSUBJECT.EXACT("Sexual harassment") OR MAINSUBJECT.EXACT("Sexual abuse") OR MAINSUBJECT.EXACT("Traumatic grief") OR MAINSUBJECT.EXACT("Neglect") OR MAINSUBJECT.EXACT("Death") OR MAINSUBJECT.EXACT("Grief") OR MAINSUBJECT.EXACT("Bereavement") OR MAINSUBJECT.EXACT("Drug abusers") OR MAINSUBJECT.EXACT("Drug addiction") OR MAINSUBJECT.EXACT("Drug addicts") OR MAINSUBJECT.EXACT("Drug dependency units") OR MAINSUBJECT.EXACT("Overdoses") OR MAINSUBJECT.EXACT("Alcohol abuse") OR MAINSUBJECT.EXACT("Substance abuse disorders") OR MAINSUBJECT.EXACT("Drug abuse") OR MAINSUBJECT.EXACT("Substance abuse")) OR (MAINSUBJECT.EXACT("Injuries") OR MAINSUBJECT.EXACT("Chronic posttraumatic stress disorder") OR MAINSUBJECT.EXACT("Psychological trauma") OR MAINSUBJECT.EXACT("Repetitive trauma") OR MAINSUBJECT.EXACT("Traumatic life events") OR MAINSUBJECT.EXACT("Vicarious trauma") OR MAINSUBJECT.EXACT("Alcohol related trauma") OR MAINSUBJECT.EXACT("Traumatic grief") OR MAINSUBJECT.EXACT("Posttraumatic stress disorder") OR MAINSUBJECT.EXACT("Traumatic stress") OR MAINSUBJECT.EXACT("Traumatic incidents") OR MAINSUBJECT.EXACT("Combat related posttraumatic stress disorder") OR MAINSUBJECT.EXACT("Physical trauma") OR MAINSUBJECT.EXACT("Violence") OR MAINSUBJECT.EXACT("Domestic violence") OR MAINSUBJECT.EXACT("Sexual violence") OR MAINSUBJECT.EXACT("Rape") OR MAINSUBJECT.EXACT("Family breakdown") OR MAINSUBJECT.EXACT("Gangs") OR MAINSUBJECT.EXACT("Harassment") OR MAINSUBJECT.EXACT("Sexual harassment") OR MAINSUBJECT.EXACT("Sexual abuse") OR MAINSUBJECT.EXACT("Traumatic grief") OR MAINSUBJECT.EXACT("Neglect") OR MAINSUBJECT.EXACT("Death") OR MAINSUBJECT.EXACT("Grief") OR MAINSUBJECT.EXACT("Bereavement") OR MAINSUBJECT.EXACT("Drug abusers") OR MAINSUBJECT.EXACT("Drug addiction") OR MAINSUBJECT.EXACT("Drug addicts") OR MAINSUBJECT.EXACT("Drug dependency units") OR MAINSUBJECT.EXACT("Overdoses") OR MAINSUBJECT.EXACT("Alcohol abuse") OR MAINSUBJECT.EXACT("Substance abuse disorders") OR MAINSUBJECT.EXACT("Drug abuse") OR MAINSUBJECT.EXACT("Substance abuse"))) OR (ab(trauma* OR rape OR "sexual haras*" OR "traumatic event" OR "adverse life events" OR "family breakdown" OR abuse OR "substance use" OR "substance misuse" OR "substance abuse" OR drug*) OR ti(trauma* OR rape OR "sexual haras*" OR "traumatic event" OR "adverse life events" OR "family breakdown" OR abuse OR "substance use" OR "substance misuse" OR "substance abuse" OR drug*))) AND (((MAINSUBJECT.EXACT("Personality disorders") OR MAINSUBJECT.EXACT("Behaviour disorders") OR MAINSUBJECT.EXACT("Mental health") OR MAINSUBJECT.EXACT("Psychiatric disorders") OR MAINSUBJECT.EXACT("Anxiety disorders") OR MAINSUBJECT.EXACT("Psychotic mood disorders") OR MAINSUBJECT.EXACT("Schizophrenia") OR MAINSUBJECT.EXACT("Mental illness") OR MAINSUBJECT.EXACT("Drug abusers") OR MAINSUBJECT.EXACT("Drug addiction") OR MAINSUBJECT.EXACT("Drug addicts") OR MAINSUBJECT.EXACT("Drug dependency units") OR MAINSUBJECT.EXACT("Overdoses") OR MAINSUBJECT.EXACT("Alcohol abuse") OR MAINSUBJECT.EXACT("Substance abuse disorders") OR MAINSUBJECT.EXACT("Drug abuse") OR MAINSUBJECT.EXACT("Substance abuse")) OR (MAINSUBJECT.EXACT("Personality disorders") OR MAINSUBJECT.EXACT("Behaviour disorders") OR MAINSUBJECT.EXACT("Mental health") OR MAINSUBJECT.EXACT("Psychiatric disorders") OR MAINSUBJECT.EXACT("Anxiety disorders") OR MAINSUBJECT.EXACT("Psychotic mood disorders") OR MAINSUBJECT.EXACT("Schizophrenia") OR MAINSUBJECT.EXACT("Mental illness") OR MAINSUBJECT.EXACT("Drug abusers") OR MAINSUBJECT.EXACT("Drug addiction") OR MAINSUBJECT.EXACT("Drug addicts") OR MAINSUBJECT.EXACT("Drug dependency units") OR MAINSUBJECT.EXACT("Overdoses") OR MAINSUBJECT.EXACT("Alcohol abuse") OR MAINSUBJECT.EXACT("Substance abuse disorders") OR MAINSUBJECT.EXACT("Drug abuse") OR MAINSUBJECT.EXACT("Substance abuse"))) OR (ab("substance use" OR "substance abuse" OR "substance misuse" OR "mental health" OR "mental ill health" OR "post traumatic stress" OR drug OR PTSD) OR ti("substance use" OR "substance abuse" OR "substance misuse" OR "mental health" OR "mental ill health" OR "post traumatic stress" OR drug OR PTSD))) AND (((MAINSUBJECT.EXACT("Qualitative data") OR MAINSUBJECT.EXACT("Qualitative methods") OR MAINSUBJECT.EXACT("Qualitative analysis") OR MAINSUBJECT.EXACT("Qualitative research") OR MAINSUBJECT.EXACT("Content analysis") OR MAINSUBJECT.EXACT("Observational research") OR MAINSUBJECT.EXACT("Interpretative phenomenological analysis") OR MAINSUBJECT.EXACT("Discourse analysis") OR MAINSUBJECT.EXACT("Multimethod research") OR MAINSUBJECT.EXACT("Focus group interviews") OR MAINSUBJECT.EXACT("Interviews") OR MAINSUBJECT.EXACT("Grounded theory") OR MAINSUBJECT.EXACT("Phenomenology") OR MAINSUBJECT.EXACT("Action research") OR MAINSUBJECT.EXACT("Ethnography") OR MAINSUBJECT.EXACT("Narratives") OR MAINSUBJECT.EXACT("Semistructured interviews")) OR (MAINSUBJECT.EXACT("Qualitative data") OR MAINSUBJECT.EXACT("Qualitative methods") OR MAINSUBJECT.EXACT("Qualitative analysis") OR MAINSUBJECT.EXACT("Qualitative research") OR MAINSUBJECT.EXACT("Content analysis") OR MAINSUBJECT.EXACT("Observational research") OR MAINSUBJECT.EXACT("Interpretative phenomenological analysis") OR MAINSUBJECT.EXACT("Discourse analysis") OR MAINSUBJECT.EXACT("Multimethod research") OR MAINSUBJECT.EXACT("Focus group interviews") OR MAINSUBJECT.EXACT("Interviews") OR MAINSUBJECT.EXACT("Grounded theory") OR MAINSUBJECT.EXACT("Phenomenology") OR MAINSUBJECT.EXACT("Action research") OR MAINSUBJECT.EXACT("Ethnography") OR MAINSUBJECT.EXACT("Narratives") OR MAINSUBJECT.EXACT("Semistructured interviews"))) OR (ab(Interview OR "grounded theory" OR ethnography OR phenomenolo* OR "focus group" OR "content analysis" OR "thematic analysis" OR "constant comparative" OR "participant observation" OR "qualitative" OR "mixed method*" OR perceive OR perception OR perspective OR view OR experience OR attitude OR belief OR opinion OR feel OR know OR understand OR explore OR elicit) OR ti(Interview OR "grounded theory" OR ethnography OR phenomenolo* OR "focus group" OR "content analysis" OR "thematic analysis" OR "constant comparative" OR "participant observation" OR "qualitative" OR "mixed method*" OR perceive OR perception OR perspective OR view OR experience OR attitude OR belief OR opinion OR feel OR know OR understand OR explore OR elicit)))

*Results 420*

**Cochrane library**

[**https://www.cochranelibrary.com**](https://www.cochranelibrary.com)

|  | MeSH descriptor: [Homeless Persons] this term only | **496** |
| --- | --- | --- |
|  | homeless* OR undomiciled OR houseless OR unhoused OR vagran* OR "street person" OR "street people" | **1309** |
|  | (rough NEXT sleep*) | **3** |
|  | sleep* NEAR/3 rough | **7** |
|  | (temp* NEXT accommodation) | **12** |
|  | (temp* NEXT shelter*) | **6** |
|  | (sofa NEXT surf*) | **1** |
|  | “risk of eviction” | **1** |
|  | MeSH descriptor: [Halfway Houses] this term only | **19** |
|  | Hostel* | **139** |
|  | (night NEAR/3 shelter*) | **11** |
|  | (winter NEAR/4 shelter*) | **0** |
|  | "emergency accommodation" | **3** |
|  | "support accommodation" | **2** |
|  | "no fixed abode" | **5** |
|  | "hard to reach" | **420** |
|  | "supported lodging" | **0** |
|  | (housing NEAR/3 instability) | **67** |
|  | (housing NEAR/3 insecurity) | **75** |
|  | (housing NEAR/3 loss) | **15** |
|  | #1 OR #2 OR #3 OR #4 OR #5 OR #6 OR #7 OR #8 OR #9 OR #10 OR #11 OR #12 OR #13 OR #14 OR #15 OR #16 OR #17 OR #18 OR #19 OR #20 | **1995** |
|  | MeSH descriptor: [Wounds and Injuries] explode all trees | **38152** |
|  | Trauma* | **41973** |
|  | MeSH descriptor: [Psychological Trauma] explode all trees | **150** |
|  | Abuse | **17368** |
|  | MeSH descriptor: [Violence] this term only | **749** |
|  | MeSH descriptor: [Gun Violence] this term only | **7** |
|  | MeSH descriptor: [Domestic Violence] this term only | **215** |
|  | MeSH descriptor: [Intimate Partner Violence] this term only | **419** |
|  | MeSH descriptor: [Physical Abuse] this term only | **39** |
|  | MeSH descriptor: [Rape] this term only | **173** |
|  | violence OR rape OR (sexual NEXT harass*) OR (traumatic NEXT event*) OR neglect OR "family breakdown" OR "substance misuse" OR "substance abuse" OR "substance use" OR "serious accident" OR drug* | **778375** |
|  | MeSH descriptor: [Sexual Harassment] this term only | **34** |
|  | MeSH descriptor: [Substance-Related Disorders] this term only | **5404** |
|  | MeSH descriptor: [Substance-Related Disorders] explode all trees | **20936** |
|  | MeSH descriptor: [Family Separation] this term only | **4** |
|  | MeSH descriptor: [Death] this term only | **393** |
|  | MeSH descriptor: [Grief] this term only | **217** |
|  | #22 OR #23 OR #24 OR #25 OR #26 OR #27 OR #28 OR #29 OR #30 OR #31 OR #32 OR #33 OR #34 OR #35 OR #36 OR #37 OR #38 | **835659** |
|  | MeSH descriptor: [Mental Health] this term only | **3217** |
|  | MeSH descriptor: [Substance-Related Disorders] this term only | **5404** |
|  | MeSH descriptor: [Substance-Related Disorders] explode all trees | **20936** |
|  | MeSH descriptor: [Anxiety Disorders] explode all trees | **10123** |
|  | "substance misuse" OR "substance abuse" OR "substance use" OR "mental ill health" OR "mental health challenges" OR psychosis OR PTSD OR "post traumatic stress disorder" OR drug* | **781232** |
|  | MeSH descriptor: [Depression] this term only | **17992** |
|  | MeSH descriptor: [Stress Disorders, Post-Traumatic] this term only | **4030** |
|  | MeSH descriptor: [Personality Disorders] explode all trees | **1833** |
|  | MeSH descriptor: [Schizophrenia] explode all trees | **9853** |
|  | #40 OR #41 OR #42 OR #43 OR #44 OR #45 OR #46 OR #47 OR #48 | **810352** |
|  | MeSH descriptor: [Qualitative Research] this term only | **2282** |
|  | perceive OR perception OR perspective OR view OR experience OR attitude OR belief OR opinion OR feel OR know OR understand OR explore OR elicit | **235054** |
|  | Qualitative | **24491** |
|  | (Mixed NEXT method*) | **5938** |
|  | Interview or "grounded theory" or ethnography or phenomenolog* or "focus group" or "content analysis" or "thematic analysis" or "constant comparative" or "participant observation" | **36246** |
|  | #50 OR #51 OR #52 OR #53 OR #54 | **267581** |
|  | #21 AND #39 AND #49 AND #55 | **439** |
